# Characterization of Differentially Expressed miRNAs and Their Predicted Target Transcripts during Smoltification and Adaptation to Seawater in Head Kidney of Atlantic Salmon

**DOI:** 10.3390/genes11091059

**Published:** 2020-09-08

**Authors:** Alice Shwe, Tone-Kari Knutsdatter Østbye, Aleksei Krasnov, Sigmund Ramberg, Rune Andreassen

**Affiliations:** 1 Department of Life Science and Health, Faculty of Health Sciences, OsloMet‒Oslo Metropolitan University, N-0130 Oslo, Norway; aliceshw@oslomet.no (A.S.); sigmundr@oslomet.no (S.R.); 2 Nofima (Norwegian Institute of Food, Fisheries and Aquaculture Research), Postboks 210, NO-1431 Ås, Norway; tone-kari.ostbye@nofima.no (T.-K.K.Ø.); Aleksei.Krasnov@Nofima.no (A.K.)

**Keywords:** smoltification, parr-smolt transformation, miRNA, seawater transfer, seawater adaptation, Atlantic salmon, head kidney, sequencing, microarray

## Abstract

Smoltification and early seawater phase are critical developmental periods with physiological and biochemical changes in Atlantic salmon that facilitates survival in saltwater. MicroRNAs (miRNAs) are known to have important roles in development, but whether any miRNAs are involved in regulation of gene expression during smoltification and the adaption to seawater is largely unknown. Here, small RNA sequencing of materials from head kidney before, during smoltification and post seawater transfer were used to study expression dynamics of miRNAs, while microarray analysis was applied to study mRNA expression dynamics. Comparing all timepoints, 71 miRNAs and 2709 mRNAs were identified as differentially expressed (DE). Hierarchical clustering analysis of the DE miRNAs showed three major clusters with characteristic expression changes. Eighty-one DE mRNAs revealed negatively correlated expression patterns to DE miRNAs in Cluster I and III. Furthermore, 42 of these mRNAs were predicted as DE miRNA targets. Gene enrichment analysis of negatively correlated target genes showed they were enriched in gene ontology groups hormone biosynthesis, stress management, immune response, and ion transport. The results strongly indicate that post-transcriptional regulation of gene expression by miRNAs is important in smoltification and sea water adaption, and this study identifies several putative miRNA-target pairs for further functional studies.

## 1. Introduction

Atlantic salmon (*Salmo salar*) are anadromous which means that they spawn, hatch and grow in fresh water, and then migrate to the sea where they spend most of their time to grow and become sexually mature [1]. Prior to migration to seawater, the young salmon undergo a process known as smoltification or parr-smolt transformation which is triggered by environmental stimuli such as photoperiod and water temperature [2]. In wild, smoltification is stimulated by increasing day-length during spring [3]. In domesticated Atlantic salmon, on the other hand, smoltification is induced by manipulation of photoperiod such as the use of dynamic artificial light regimes [4,5]. Smoltification stimulates changes in the biochemistry, physiology, morphology, and behavior of the young salmonids [6,7]. A number of hormones including growth hormone, cortisol, thyroid hormone, prolactin, insulin and sex steroids change during smoltification [8,9,10,11,12,13,14]. Together, these changes affect the major osmoregulatory organs such as gill, gut and kidney which allow smoltified fish to initiate a successful transition from life in freshwater to seawater [15]. Anadromous salmonids are ready for seawater transfer (SWT) only when they have undergone smoltification. A fully completed smoltification is therefore crucial for the young salmon to survive in the saltwater environment [16].

Atlantic salmon is one of the major species produced in world aquaculture [17]. Over the years, the development of new technological solutions and research in aquaculture contributed to tackle challenges in salmon farming. However, the loss of farmed salmon between sea-transfer and harvest remains high. According to the annual summary of fish health in Norway [18] from the Norwegian Veterinary Institute, the mortality post seawater transfer of Norwegian farmed salmon in 2018 was 46 million which corresponds to a mortality rate of approximately 15%.

The first month post SWT is an important and critical period in the life of farmed Atlantic salmon [7]. Previous studies reported that a major portion of losses takes place in the first months after sea transfer [19,20]. The high mortality rate of farmed salmon in this period of seawater phase represents a major fish health problem as well as a major economic challenge for the aquaculture industry. The post SWT phase mortality is commonly associated with fish being vulnerable to viral pathogen, bacterial and parasitic infectious diseases [18,21,22]. A consequence of the current management routines is that some of the fish are incompletely smoltified, and this also lead to increased mortality in the post SWT period [18,19]. Many studies have been carried out on smoltification focusing mainly on study of physiology [2], endocrinology [9,23,24], osmoregulation [25], and behavior [26]. In recent years, a few studies have reported how mRNA expression is modulated during smoltification [27,28].

MicroRNAs (miRNAs) are small single stranded RNA molecules of approximately 22 nucleotides (nts) that guide the mRNA silencing complex (RISC) to the target transcripts (binding usually to the 3′UTR of the target transcript) [29]. The biologically active mature miRNAs are produced by a cascade of intranuclear and cytoplasmic enzymatic processing steps. Finally, usually one (the guide miRNA) out of the two duplex miRNAs (5p or 3p) from Dicer processing of the pre-miRNA is incorporated with Argonaute into the RISC complex while the other (the passenger miRNA) is ejected and degraded [30]. The RISC complex with its guide miRNA regulate gene expression by degrading or repressing translation of target mRNAs at the post-transcriptional level [29,30]. Expression analysis usually show there is a difference in abundance of the two mature miRNAs (5p and 3p) originating from a miRNA precursor. This is assumed to be a consequence of the final step of the miRNA biogenesis where the guide miRNAs (either 5p or 3p) is loaded onto Argonaute while the passenger miRNA is degraded [29,30]. Consequently, one may assume that the more abundant mature miRNA originating from a precursor in most cases is the guide miRNA. Due to the salmonid specific genome duplication the number of miRNA genes in miRNA gene families is often twice as high as in other teleost (e.g., the miRNA-194 family have 4 members leading to 8 mature miRNAs (5p and 3p)) [31,32]. However, it is often a large difference in expression between these family members with one member being highly expressed. One would assume that it is the highly expressed family member that is the biologically most important mature miRNA from this family. We therefore find it meaningful to refer to the assumed guide miRNA from the highly expressed gene member of a miRNA gene family as the major expressed mature miRNA from the family (major expressed miRNA). It has been demonstrated that a single miRNA may regulate many mRNA transcripts (target genes) and a single mRNA may be regulated by several distinct miRNAs [33]. A number of studies have reported that miRNAs are involved in many biological processes including developmental transition, growth, tissue differentiation, immune response, and response to environmental changes [34,35,36,37,38]. At present, there have been identified and annotated 589 different mature miRNAs in Atlantic salmon [31,32]. In lack of recent updates of miRbase (v.22.1 is the most recent, http://www.mirbase.org/), only the 472 miRNAs from the first identification study [31] are in the present version of miRbase. A study by Woldemariam et al. (2019) [32] characterized miRNAs that were highly expressed in certain tissues indicating that they have organ specific functions. A smaller group of miRNAs have also been reported to be associated with immune responses in challenge trials indicating that they have important roles in the immune system responses [38,39,40,41] or in maturation of macrophages [42]. Whether miRNAs are involved in regulation of gene expression during smoltification and whether they facilitate adaption to seawater is largely unknown.

The aim of this study was, therefore, to characterize miRNAs associated with smoltification and the adaptation to seawater in the first month post SWT period. In addition to a characterization of the miRNA expression changes, the mRNA expression changes were investigated in the same samples. The study was carried out in materials from head kidney, as in Atlantic salmon, this is an organ with important immune, endocrine, and osmoregulatory functions [43,44,45]. Knowledge on the post transcriptional interaction between miRNAs and their target mRNAs during these critical periods of Atlantic salmon life is essential when aiming to understand how fine-tuning of gene expression may facilitate this important developmental transition. Such knowledge also has the potential to aid in changes on today’s management to improve fish health and reduce the mortality post SWT in aquaculture.

## 2. Materials and Methods

### 2.1. Ethical Statement

All fish handling procedures complied with the Guidelines of the EU-legislation (2010/63/EU), as well as with the Norwegian legislation. The experiment is considered as non-regulated procedure according to the National Legislation on Animal Research since the fish had not been exposed to any pain or distress. The fish were solely killed for the use of their tissues for research. Thus, the experiment did not require application for approval from the Norwegian Food Safety Authority.

### 2.2. Fish and Sampling

The experimental fish trial was carried out over a period of 16 weeks (from July to October 2018) at the Nofima’s Research Station for Sustainable Aquaculture (Sunndalsøra, Norway). A total of 70 Atlantic salmon from SalmonBreed strain were used. The fish were kept in one tank supplied with running water throughout the experiment and they were fed commercial dry feed (Skretting, Norway). From start feeding, the fish were kept under ordinary production conditions in freshwater with average water temperature at 13 °C and 24 h continuous light. Two weeks before the start of light treatment, the average water temperature dropped to 8 °C. Subsequently, the initiation of light treatment (Supplementary Materials Figure S1) started by decreasing daylight from 24 h to 12 h. At the same time the water temperature increased from 8 °C to 13 °C for 5 days, followed by 12 °C for 41 days. The fish were then subjected to 24 h light again for final stage of smoltification process. Both seawater challenge test [46,47], plasma chloride level test and the change to silvery coloration of the skin showed that the experimental fish were optimally smoltified 81 days after onset of the experiment, and were, therefore, transferred to seawater. The average weight at this time point was 72.4 ± 8.7 g.

Samples for miRNA and mRNA expression profiling were collected at six time-points (Supplementary Materials Figure S1). The sampling points were T1: one day prior to light treatment, T2: halfway through light treatment (46 days post onset of light treatment (POL)), T3: three quarters into the light treatment period (67 days POL), T4: one day prior to SWT (81 days POL), T5: one week after SWT (88 days POL), and T6: one month after SWT (111 days POL). The experimental conditions such as day light, water temperature, average weight of experimental fish and water type at each sampling points are provided in Table 1. At each time points, 10 fish were anesthetized by an overdose of MS-222 (tricaine methanesulfonate, 0.1 g/L) prior to weighing and sampling. The fish were then killed by a blow to the head and the tissue samples for gene expression analyses were collected from head kidney. The samples were frozen immediately in liquid hydrogen and stored at −80 °C.

### 2.3. Total RNA Extraction

Total RNA including the small RNA fraction were isolated from head kidney samples using mirVanaTM miRNA Isolation Kit (Ambion, Life Technologies, Carlsbad, CA, USA) according to the manufacturer’s protocol. RNA concentrations and purity were determined using NanoDropTM1000 Spectrophometer (Nanodrop ND-1000, Thermo Fisher Scientific, Wilmington, DE, USA). The integrity of total RNA (RIN value) was assessed using the Agilent 2100 Bioanalyzer in combination with an Agilent 6000 Nano Chip (Agilent Technologies, Santa Clara, CA, USA). The extracted RNA was stored immediately at −80 °C.

### 2.4. Small-RNA Sequencing

A total of 48 RNA samples from head kidney were sequenced. There were 8 samples from each of the time points T1–T6. The small-RNA libraries for the 48 samples were constructed by use of NEBnext^®^ multiplex small RNA Library Prep Set (New England Biolabs, Inc., Ipswich, MA, USA) in accordance with manufacturers protocol. One µg of total RNA from each of the samples was used as input for preparation of the libraries that included 5′ and 3′ adapter ligation, reverse transcription, PCR amplification and size selection of 140–150 bp fragments using 6% polyacrylamide gel. The sequencing was performed on an Illumina NextSeq 500 providing 75 bp single end reads. Both library preparation and sequencing were performed at the Norwegian Sequencing Centre (NSC). All sequenced samples have been submitted to the NCBI Sequence Read Archieve (SRA) (https://www.ncbi.nlm.nih.gov/sra/) with accession bioproject number PRJNA556577.

### 2.5. Pre-Processing, Quality Control and Analysis of Small-RNA Sequencing Data

FASTQC software (v.0.11.8) (http://www.bioinformatics.babraham.ac.uk/projects/fastqc/) was applied for quality control of raw sequence reads (fastq-files) to ensure the data was of good quality for downstream analysis.

The adapter sequence (5′ AGATCGGAAGAGCACACGTCTGAACTCCAGTCAC 3′) were removed followed by a size filtering of the raw reads with Cutadapt (v.2.3) Python Package (v.3.7.3) [48]. Reads that were shorter than 18 nts or longer than 25 nts were discarded. Additional FASTQC analysis of trimmed reads after size filtering was performed to ensure that the per base sequence quality showed phred scores of 32 or more in all samples.

The adapter trimmed and size filtered reads from each of the 48 samples were mapped to a reference index of all known *Salmo salar* mature miRNAs [32] using STAR aligner software (v.2.5.2b) [49]. Next, the output files of STAR mapping (BAM format) were processed further in R-Studio by using the feature Counts function from the Rsubread package (v.1.34.2) to produce count matrices [50]. The count tables were used as input in the DESeq2 R package (v.1.24.0) for differential expression analysis by comparing each of the time points T2, T3, T4, T5 and T6 with T1. A Benjamini-Hochberg adjusted *p*-value ≤ 0.05, log2 fold-change with absolute value ≤ −1.0 or ≥1.0, and basemean read counts ≥10 were applied as threshold to identify differentially expressed (DE) miRNAs. The DE miRNAs that fulfilled threshold requirements were extracted and subjected to hierarchical clustering using Heatmap2 from R-package gplots (v.3.0.1.1). The optimal number of clusters for k-means clustering was determined using Gap statistic method from R-package factoextra (v.1.0.5). Spearman correlation and Complete linkage method were used for hierarchical clustering analysis.

### 2.6. Microarray Analyses and Gene Ontology Enrichment Analysis

The mRNA expression analysis was carried out on the Atlantic salmon 44 k DNA oligonucleotide microarray containing 60-mer probes to all identified protein coding genes (Salgeno-2, GPL28080), designed at Nofima and annotated with bioinformatic package STARS [51]. Microarrays were manufactured by Agilent Technologies (Inc., Cedar Creek, TX, USA), and the reagents and equipment were from the same source. One-color hybridization was employed, and each sample was analyzed with a separate array. Totally, 29 of the 48 fish selected for small-RNA Sequencing were included representing 6 time points (T1–T6), with 5 fish per time point except T5 where 4 fish were analyzed.

RNA was diluted to 100 ng/µL and 2.2 µL of each sample was used for cDNA synthesis, amplification and Cy3 labeling of cRNA using LowInput QuickAmp Labeling Kit according to the manufacturer’s protocol. The labeled/amplified cRNA (Cy3-cRNA) were purified using Qiagen’s RNeasy Mini Kit (QIAGEN group, Hilden, Germany) and quantified to determine the specific activity. In total, 1650 ng of Cy3-labeled cRNA was used to prepare hybridization mix for each sample using Gene Expression Hybridization Kit. Next, the hybridization was carried out at 65 °C for 17 h with rotation speed at 10 rpm in a hybridization oven. Finally, the arrays were washed with Gene Expression Wash Buffer 1 and 2 and scanned with SureScan Microarray Scanner (Agilent Technologies, Santa Clara, CA, USA). Nofima’s bioinformatic package STARS [51] was used for data processing. The default parameters of this package were used; a threshold of log2-foldchange ≤ −0.80 or ≥0.80 and *p* ≤ 0.05 were used to identify significant differentially expressed genes (DEG). DEGs were identified by comparing each of the time points (T2–T6) with T1 (before the onset of smoltification).

Next, the expression of mRNAs and miRNAs were compared. The miRNAs with unnormalized read counts > 10 in at least 2/3 samples through the time points investigated were grouped with K-mean clustering. Correlation between the mean expression profiles of the clustered miRNAs and the expression profiles of mRNA was analyzed. Clusters and DEG with r > 0.67 or <−0.67 were annotated as correlated expression profiles (positively or negatively correlated to the expression of the DE miRNAs).

The gene ontology enrichment analysis of negatively correlated genes was carried out using PANTHER Statistical Overrepresentation Test (Released 2020-04-07) with GO database (Released 2020-03-23) for dataset of GO biological process and GO cellular component, and Reactome pathway dataset (v.65 Released 2019-12-22) for pathway analysis (http://pantherdb.org) [52]. Some gene symbols from the microarray annotations on the Atlantic salmon 44k array were converted to the human ortholog symbols for practical reasons. The genes (*n* = 81) with negatively correlated expression to the DE miRNAs were analyzed regarding enrichment of certain GO’s for biological process and cellular location using Danio rerio as reference. This provided enriched sets of genes that shared GO groups. Fisher’s exact test was used for estimates of significance, and *p*-values were adjusted with Benjamini–Hochberg method. A false discovery rate (FDR) less than 0.05 were considered as significant. Applying the Hierarchy option, the output was sorted in a hierarchical system giving groups sharing a very general process on top and more specific functions within the general GO. For this reason, the genes given in Supplementary Materials Table S1 are in some cases grouped in GO of more general terms and then additionally grouped in GO’s that are more specific within the general GO term. The UniProt knowledgebase (https://www.uniprot.org) was used as an alternative source to obtain gene ontology (GO) terms of some of the negatively correlated genes. Finally, the negatively correlated genes were mapped to Reactome pathways to identify the most enriched gene pathways.

### 2.7. In Silico Prediction of miRNA Target Genes

The miRNA target prediction tool RNAhybrid (v.2.2) [53] was applied to identify the putative target genes of the 71 DE miRNAs using their mature sequences and the 3′UTRs of the 2472 DE genes identified in the microarray analysis as input in the prediction analysis. The 3′ UTR sequences were retrieved from NCBI Reference Sequence database (Refseq; https://www.ncbi.nlm.nih.gov/refseq/). The 3′ UTR sequences from 60 genes were retrieved from our own full-length sequenced dataset obtained from the same materials. Two hundred and thirty-seven DE genes could not be included in the in silico analysis as 3′UTR sequences were not available in GenBank or our own full length sequenced dataset. The following parameters were applied in the RNA hybrid analysis: No G:U in seed, helix constraint 2–8, loop constraints 9–9 and a minimum free energy threshold of ≤−18 kcal/mol. These parameters allowed RNAhybrid to detect only candidate genes with perfect seed complementarity and high base-paring stability.

## 3. Results

### 3.1. RNA Extraction and Small-RNA Sequencing

Total RNA was extracted from 48 head kidney samples collected before, during smoltification and post SWT (Table 1 and Supplementary Materials Figure S1). The concentration of total RNA in extracted samples ranged from 135 to 242 ng/μL and all RIN values were above 8.0. Individual sample concentrations, RIN values and absorbance ratios are given in Supplementary Materials Table S2. All samples were used for small RNA sequencing and individual small RNA libraries were successfully prepared and sequenced. The results from RNA extraction, the number of raw reads, the number of adapters trimmed and size filtered reads as well as reads mapped uniquely as miRNAs for each sample are given in Supplementary Materials Table S2. The total number of reads obtained from the small-RNA sequencing ranged from 1.3 million to 9 million. The size-filtered and adapter trimmed reads uniquely mapped as mature *Salmo salar* miRNAs for all samples ranged from 255972 to 2734599, and the average size of these reads were 22 nts. Raw FASTQ files from all sequenced samples have been submitted to the Sequence Read Archive (SRA) in NCBI with bioproject number PRJNA556577, and accession numbers for each sample are given in Supplementary Materials Table S2. Length distribution for each sample after adapter trimming and size filtering is given in Supplementary Materials Figure S2.

### 3.2. Identification of DE miRNAs in Atlantic Salmon Head Kidney during Smoltification and Post SWT

DESeq2 analyses were applied to identify significant differentially expressed (DE) miRNAs (Benjamini-Hochberg adjusted *p*-value ≤ 0.05, log2 fold-change ≤ −1.0 or ≥1.0 and basemean read counts > 10) in the head kidney of Atlantic salmon by comparing the expression immediately before smoltification, during smoltification and post SWT (Table 1 and Supplementary Materials Figure S1). A total of 71 miRNAs revealed significant differences in at least one of the six time points compared. The DE miRNAs belonged to 45 miRNA families including three miRNAs only discovered in Atlantic salmon (novel-2, novel-12, and novel-15) [32]. A complete summary of the relative expression changes of the 71 miRNAs at all time points are provided in Supplementary Materials Table S3.

**Figure 1:**
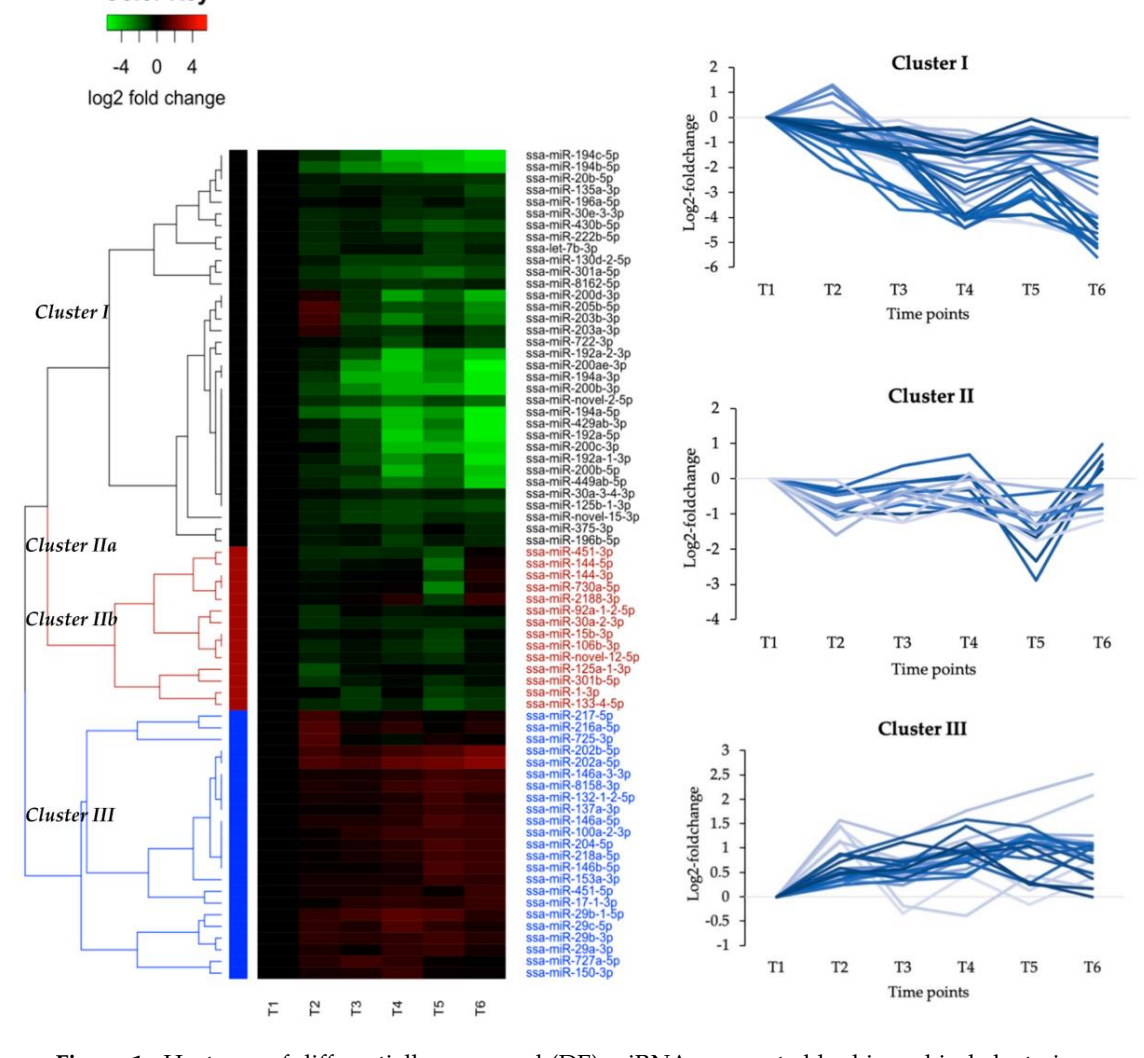
Heatmap of differentially expressed (DE) miRNAs generated by hierarchical clustering. All expression at T2–T6 is relative to T1 (pre-smolt, one day before smoltification). T2–T4 are relative expression during light treatment period/smoltification while T5–T6 are relative expression in the post SWT period. Expression at each time point (columns) is based on independent small RNA sequencing of 8 samples. Each row in the heatmap represents the relative expression of the mature miRNA annotated to the right for the row. The magnitude and direction of expression changes illustrated by color is given by the color key above the heatmap. Each of the three major clusters is denoted by color codes on the left side of the heatmap (Cluster I-black, Cluster II-red, Cluster III-blue). On the right side of the heatmap, additional plots illustrate the expression profiles of each cluster. The relative log2 foldchange compared to T1 is illustrated for other time points. Graphs highlighted in dark blue indicate most typical expression profile of each cluster while graphs highlighted in light blue correspond to less characteristic expression profiles.

Hierarchical clustering analysis of the 71 DE miRNAs showed three major clusters that differed in their expression profiles (Figure 1). In general, miRNA members from the same family revealed same expression patterns and clustered together. Cluster I contained a relatively large group of 34 mature miRNAs that belonged to 22 different miRNA families. The major expressed mature miRNAs (see definition in introduction) in Cluster I are shown in Table 2 (No. 1–23). The families with largest number of mature miRNA members were miRNA-192, miRNA-194 and miRNA-200. The expression pattern common to the miRNAs in this cluster was a down regulation at most time points compared to T1 (Figure 1, Cluster I). The three mature miRNAs from the miRNA-203 family, miR-200d-3p and miR-205b-5p showed a slight deviation from others with an increased expression at T2. However, at all later time points they were, like the others, downregulated compared to T1. Also, a temporary increase in expression at one-week post seawater transfer (T5) compared to expression immediately before SWT (T4) was observed for the major part of these miRNAs (additional plot, Cluster I, Figure 1), but the decreased expression was maintained at one-month post SWT (T6).

Cluster II consisted of 14 miRNAs that belonged to 13 different miRNA families. The major expressed ones from each family in this cluster are shown in Table 2 (No. 24–37). The expression pattern common to the miRNAs in this cluster was a decrease in their expression that was most pronounced at one-week post SWT (T5). Five miRNAs that belonged to the family of miRNA-451, miRNA-144, miRNA-730 and miRNA-2188 clustered together within Cluster II (Figure 1, Cluster IIa). These miRNAs showed a large decrease at one-week post SWT (T5) that was followed by a large increase at one-month post SWT (T6).

Cluster III included 23 miRNAs that belonged to 17 different miRNA families. The major expressed members of Cluster III are shown in Table 3. The miRNA families miRNA-202, miRNA-146, and miRNA-29 were the ones with most numerous members. The expression changes common to the Cluster III miRNAs were an increase in their expression through the experimental period (T2–T6). Also, the two miRNA-202 family members revealed a particularly large increase at T5 and T6.

### 3.3. Identification of DE mRNAs and a Subset of DE mRNAs with Negative Correlation to the DE miRNAs

The expression changes of protein coding genes in Atlantic salmon head kidney during smoltification and post SWT were investigated by microarray analysis. The material and time-points were the same as the ones analyzed for characterization of miRNA expression. The analysis revealed 2709 mRNAs that were differentially expressed in at least one of the time points compared (log2 fold-change ≤ −0.80 or ≥0.80 and *p* ≤ 0.05). A complete overview of all these DE genes along with their expression changes over the experimental period and *p*-values are provided in Supplementary Materials Table S4.

DE mRNAs that were negatively correlated to DE miRNAs were identified by correlation analyses of the mean expression profiles from the clustered miRNAs against the expression profiles of the DE mRNAs. A total of 11 (group A) and 70 (group B) out of the 2709 DE mRNAs showed such negative correlation to miRNAs in Cluster I and III, respectively. A complete overview of these 81 genes including gene ontology annotations is given in Supplementary Materials Table S5.

The group A DE mRNAs were sorted based on their functional similarity (GO annotations). Steroid hormone biosynthesis was the largest group while the other six DE mRNAs were distributed on different biological functions. The 70 group B DE mRNAs, negatively correlated to Cluster III, were also sorted based on their functional similarity. The GO terms of molecular function and biological process showed transport across membrane as the largest group.

#### 3.3.1. Enrichment Analysis of Negatively Correlated Genes

**Figure 2:**
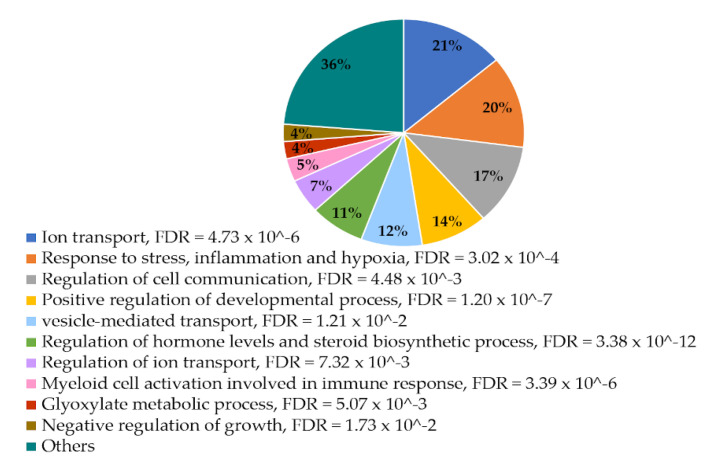
Distribution of largest groups sharing significantly enriched biological processes among the negatively correlated DE genes. Proportions are estimated from total number of negatively correlated genes for each gene ontology (GO) group, but as several genes belong to more than one group, the sum is more than 100%.

Several biological process groups were significantly enriched in the list of 81 negatively correlated mRNAs (Figure 2). Ion transport was the largest group with 17 genes (21%) while 6 genes (7%) belonged to the related group; regulation of ion transport. The second largest group was response to stress with subcategories response to hypoxia and inflammation with 16 genes (20%). Other significantly enriched groups were regulation of cell communication with 14 genes (17%), positive regulation of developmental process with 11 genes (14%), vesicle-mediated transport with 10 genes (12%), regulation of hormone levels and steroid biosynthetic process with 9 genes (11%), myeloid cell activation involved in immune response with 4 genes (5%), glyoxylate metabolic process with 3 genes (4%) and negative regulation of growth with 3 genes (4%). Twenty-nine genes were not enriched in any of these categories, thus, shown as others in Figure 2. A complete overview of all significantly over-represented GO groups and the genes in each group is provided in Supplementary Materials Table S1.

The enrichment analysis with the gene ontology terms from cellular component revealed plasma membrane as the significantly enriched cellular location (FDR = 5.54 × 10^−11^) with largest number of genes (38 genes, 47%). The second largest enriched cellular location group was mitochondrion with 12 genes (15%) and a FDR = 2.35 × 10^−3^. The remaining 38 genes were mapped to several other cellular locations. The gene pathway enrichment analysis was also carried out using the PANTHER Overrepresentation Test. There were, however, only two significantly enriched gene pathways; solute-carrier (SLC) mediated transmembrane transport with 11 genes (FDR = 5.97 × 10^−6^) and metabolism of steroid hormones with 4 genes (FDR = 5.11 × 10^−3^).

### 3.4. In Silico Target Gene Predictions

In silico prediction of target genes is a common mean to reveal what genes may be regulated by a given miRNA. A target gene prediction analysis with all 71 DE miRNAs as input was, therefore, carried out against the 3′UTRs of all the 2412 DE mRNAs that had 3′UTR sequences available from GenBank or provided from our own full-length sequenced mRNAs (see methods). This in silico analysis identified 1827 transcripts as putative target genes of the 71 DE miRNAs. An overview of all 1827 putative target genes are provided in Supplementary Materials Table S6. Among the 1827 putative target genes, 42 were in the group that were negatively correlated in expression to the DE miRNAs in Cluster I or III (Section 3.3). As target transcripts are negatively regulated by their targeting miRNAs, these genes are best candidates to be true targets. These 42 DE mRNAs and the DE miRNAs targeting these genes are further described in Section 3.4.1.

#### 3.4.1. Predicted Targets Negatively Correlated to DE miRNAs in Cluster I and III

Six predicted target genes were negatively correlated to eight of the DE miRNAs that belonged to Cluster I while 36 predicted target genes were negatively correlated to 18 of the DE miRNAs in Cluster III. Thus, 42 of the 81 negatively correlated DE mRNAs were predicted as target genes of one or more of the DE miRNAs. All the predicted targets of miRNAs in Cluster I and III are shown in Table 4 while additional information like gene name, gene ontology and accession number are given for each target in Supplementary Materials Table S7. Thirty-six out of all the 71 DE miRNAs targeted at least one gene, and most of these genes (86%) were targeted by at least one of the major expressed mature miRNAs. The results showed that, in some cases, one DE miRNA targeted several genes, e.g., ssa-miR-217-5p that targeted eight genes (No. 10, 16, 20, 26–29, 31, Table 4). On the other hand, one gene could be targeted by several of the miRNAs, e.g., Nuclear receptor-interacting protein (*nrip2*) that was targeted by six miRNAs (No. 16, Table 4).

Several of the targets were genes belonging to one of the significantly enriched biological process groups (Section 3.3.1). Four of the targets were from the enriched group; regulation of hormone level and steroid biosynthetic process (Table 5). Three of these genes (*star* paralog 1, *star* paralog 1 and *cxa1*) are essential in the steroid hormone biosynthesis. The downregulation of miRNAs that targeted these genes, all from Cluster I, would contribute to a posttranscriptional upregulation of these genes.

Eleven predicted target genes (*agtrap*, *lpl*, *pfkfb1*, *cdo1*, *cld3*, *sepp1*, *epn*, *sell*, *star paralog 1*, *star paralog 1*, and *cxa1*) were from the GO enriched groups of stress response, response to hypoxia, inflammatory response and myeloid cell activation involved in immune response (Table 6). All predicted genes in this group except the genes involved in general hormone biosynthesis (*star paralog 1*, *star paralog 1* and *cxa1*) were targeted by the DE miRNAs from Cluster III that showed an increase in expression during the smoltification and the first month after SWT.

Upregulation of DE miRNAs in Cluster III would lead to a posttranscriptional downregulation of the targets (*agtrap*, *lpl*, *pfkfb1*, *cdo1*, *cld3*, *sepp1*, *epn,* and *sell*). One of these genes, selenoprotein (*sell*) is found only in fish, not in higher vertebrates, and, interestingly, it is targeted by miR-725 which is also only present in teleosts. Selenoprotein P (*sepp1*) is another of the targets (ssa-miR-204-5p). This protein is mainly synthesized in kidney and important for transport of selenium which are essential to fish health and immunity [54,55].

Seven other predicted targets (*npt2a*, *s47a1*, *slc16a7*, *slco1a2*, *gat2*, *slc15a2,* and *spink1*) were from the enriched groups; ion transport and regulation of ion transport (Table 7). The six genes from the ion transport group were targeted by DE miRNAs in Cluster III. Upregulation of DE miRNAs in Cluster III would, therefore, lead to post-transcriptional downregulation of these genes at T5 and T6. On the other hand, *spink1*, that participates in regulation of ion transport, was targeted by two miRNAs belonged to Cluster I, thus, leading to a post-transcriptional upregulation of this transcript.

## 4. Discussion

### 4.1. Several DE miRNAs Associated with Smoltification and Early Seawater Adaptation Are Conserved miRNAs Reported as Involved in Stress Response, Hypoxia, and Immune System Functions

The current study identified miRNAs and mRNAs that were differentially expressed in head kidney before and during smoltification as well as in the early sea water phase. There were 71 miRNAs that were significantly and differentially expressed during the six time points compared. The hierarchical clustering analysis of the 71 DE miRNAs revealed three major clusters with three different expression patterns (Figure 1). The miRNA families with largest number of members in Cluster I were miRNA-192 and miRNA-194 (Table 2). Interestingly, the miRNA-192 and miRNA-194 families are clustered miRNA genes highly expressed in kidney, intestine and liver tissue of Atlantic salmon [32] indicating that they are involved in regulation of some common tissue specific functions. Furthermore, miR-192 has been reported as a deregulated miRNA in response to hypoxia [56]. This miRNA is also downregulated in liver to protect against oxidative stress induced damage in mammals [57] by upregulating *zeb2* (Zinc finger E-box-binding homeobox 2-like protein). In our predictions miR-192a-5p did not target *zeb2*, but Cell death activator CIDE-3, another gene involved in apoptosis. On the other hand, ssa-miR-125b-3p, ssa-miR-200ae-3p, ssa-miR-200b-3p, ssa-miR-and 429ab-3p, all belonging to Cluster I, did target the Atlantic salmon ortholog of *zeb2* (Supplementary Materials Table S6). All, except ssa-miR125b-3p were also predicted to target the hypoxia inducible factor which is the main regulator of cellular oxygen homeostasis [58]. A reduction of these miRNAs would result in upregulated *zeb2* and protection against stress related apoptosis. *Zeb2* is also targeted by miR-200 in other species indicating a conservation in miRNA-target interaction [59]. Among the other DE miRNAs in Cluster I both let-7b and miR-30a are responding to hypoxia in zebrafish [60] while miR-20 and miR-125 are generally deregulated in all kinds of cellular stress [56]. In addition, miR-375 are also among the Cluster I miRNAs that has been reported as responding to hypoxia [61]. All these findings suggested that many of the Cluster I miRNAs (and some Cluster II miRNAs mentioned below) could be involved in regulation of cellular oxygen homeostasis. However, we also used a more direct approach to identify the target genes of the DE miRNAs. This approach suggested that many of the Cluster I miRNAs also were involved in regulation of hormone levels (see Section 4.2).

The 14 major expressed mature miRNAs belonging to Cluster II share properties with the Cluster I miRNAs as their expression changes would lead to increases in target gene expression during smoltification and the first week post SWT. Especially, ssa-miR-451-3p, ssa-miR-144-5p, ssa-miR-144-3p, ssa-miR-730a-5p, and ssa-miR-2188-3p had large decreases in expression immediately after SWT (T5), but these miRNAs also increased to initial levels after four weeks in saltwater (T6). Ssa-miR-106b-3p from the Cluster II miRNAs was among the miRNAs predicted to target *zeb2* while ssa-miR-144-3p was predicted to target the hypoxia inducible factor. MiR-106 is reported as deregulated in response to hypoxia [56]. Two other miRNAs in this cluster, miR-1 and miR-92, have also been associated with hypoxia in zebrafish [60]. Ssa-miR-2188-3p has previously been reported to decrease its expression in Atlantic salmon infected with *Salmonid* *alphavirus* (SAV) and predicted to target genes of the immune system signaling pathway [38]. Recently, it was also suggested to be involved in macrophage maturation [42].

There were 17 major expressed mature miRNAs belonging to Cluster III (Table 3). They showed an increase in expression from the onset of smoltification and most of these miRNAs remained upregulated post SWT. The most abundant miRNA families in Cluster III were miRNA-29, miRNA-146 and miRNA-202. Both miRNA-29 and miRNA-146 families have been reported to increase their expression in response to SAV infection [38] while ssa-miR-29b-1-5p, ssa-miR-100a-2-3p, ssa-miR-132-1-2-5p, and ssa-miR-8158-3p were reported as responding to IPNV-infection ([62], In Press). Previous studies also reported that ssa-miR-29b-1-5p, ssa-miR-146a-5p, ssa-miR150-3p and ssa-miR-725-3p are involved in macrophage maturation [42]. Together, this indicates that many of the miRNAs in Cluster III, increasing their expression during smoltification and SWT, could be involved in downregulation of immune system genes. If so, this would be in accordance with susceptibility to pathogens and repression of the immune transcriptome during smoltification and first weeks of sea water period reported in several studies [28,63]. The two family members ssa-miR-202a-5p and ssa-miR-202b-5p revealed a particularly large increase at T5 and T6. MiRNA-202 have been reported as highly expressed in gonads in teleosts (e.g., [64]). However, it is also expressed in head kidney in salmonids [31,65,66]. Interestingly, this miRNA responds to heat stress in rainbow trout [66]. The function of ssa-miR-202 is unknown, but if assuming it is a common target in head kidney and gonads, we note that both gonads and head kidney are organs where there is biosynthesis of hormones. This opens the possibility that ssa-miR-202 are involved in regulation of a common target in the hormone biosynthesis pathway in these organs. The putative functions of the other miRNAs in this cluster were further explored by comparisons to our microarray results and the in silico target predictions (see below, Section 4.2).

### 4.2. Enrichment Analysis and In Silico Target Predictions Indicates that the DE miRNAs Are Ivolved in Regulation of Hormone Levels, Stress Response, and Ion Transport

The microarray analyses revealed 2709 mRNAs as significant differentially expressed during smoltification and SWT. In general, the mRNA expression changes were in accordance with prior similar transcriptome studies [27,28]. The miRNA-target predictions often result in many false positive predicted target genes [37,67]. To minimize the number of false positives, we carried out a correlation analysis to identify miRNAs and mRNAs that showed negatively correlated expression patterns over the sampling period. Any such negatively correlated DE mRNAs are more likely to be true miRNA target genes as their expression changes are in agreement with the expected results if targeted by a DE-miRNA at the post-transcriptional level [29]. Other of such negatively correlated DE genes that are not themselves predicted as targets could still be indirectly affected by the DE miRNAs, e.g., if any of the DE miRNAs affect the expression of other upstream genes in the same biological pathways. Utilizing this approach, we aimed to increase the confidence in target gene predictions and identified a smaller set of target transcripts that fit both criteria of negative proportional mRNA expression as well as being predicted as miRNA-target gene pairs. It is still possible that some target mRNAs are translationally repressed rather than degraded. However, such targets may only be identified by comparing miRNA expression with protein expression data rather than mRNA expression data. Finally, we carried out enrichment analysis using all 81 negatively correlated DE genes (including the 42 predicted target) to better understand what biological processes the DE miRNAs were likely to regulate during smoltification and the early sea water period.

Regulation of hormone levels and steroid biosynthetic process was a significantly enriched group with nine negatively correlated genes (Supplementary Materials Table S1) and four of these (*fdx1l, star* paralog1, *star* paralog2, and *cxa1*, Table 5) were predicted as targets of Cluster I miRNAs. STAR is a sterol transfer protein that regulates transport of cholesterol from the cytoplasm to the inner mitochondrial membranes, and by this, a key regulator of the production of steroid hormones from cholesterol [68]. Another of the targets was Adrenodoxin (*fdx1l*) that is involved in synthesis of cortisol [69]. Upregulation of these targets means that the level of proteins such as steroid hormones and cortisol will increase. Previous studies have reported that serum cortisol levels and hormones, like thyroid hormone are elevated during smoltification [14,63,70]. The head kidney in fish comprises the endocrine cells producing and secreting cortisol, catecholamines and thyroid hormones [45]. Here we show that miRNAs may be involved in regulation of the steroid biosynthesis in head kidney and by this, involved in regulation of hormone levels during smoltification [7,8,9,10,11,12,14].

The enrichment analysis also revealed response to stress, inflammation, hypoxia, and myeloid cell activation involved in immune response as significantly enriched groups among the negatively correlated DE genes (Supplementary Materials Table S1). Eight of the genes annotated in these groups were also predicted as targets of Cluster III miRNAs (Table 6). Common to Cluster III miRNAs were increased expression during the smoltification and first period in sea water, and the negative regulation of their targets would affect the management of cellular stress. Changes in stress levels is also associated with smoltification and sea water adaptation [63] and the results here indicate that some of the DE miRNAs are involved in management of cellular stress.

One of the largest enriched biological process groups with negatively correlated DE genes was ion transport and regulation of ion transport. In addition, plasma membrane was the largest enriched cellular location. Six of the genes in this group were targets of Cluster III miRNAs (Table 7). Moreover, aquaporin 8, a gene encoding a renal aquaporin which is important in osmoregulation [71] was targeted by the Cluster III DE miRNA ssa-miR-217-5p. Mostly, the targeted DE genes that belonged to the enriched groups of ion transport and regulation of ion transport were integral components of the plasma membrane. One target gene participating in regulation of ion transport (*spink1*) was, unlike the others, targeted by two Cluster I DE miRNAs (ssa-miR-196a/b-5p). These findings indicate that some DE miRNAs are involved in regulation of the cellular changes in plasma membrane proteins needed to tolerate ionic seawater concentrations when migrating from hypotonic to hypertonic environment.

Omics approaches often results in massive data information. Here, we first carried out transcriptomic analyses of gene expression applying a microarray platform and miRNAomic analysis of miRNA expression applying small RNA sequencing. Correlation analysis, in silico predictions of target genes and gene enrichment analysis were then used to better interpret the regulatory role of the differentially expressed miRNAs discovered. In conclusion, the results indicate that miRNAs are involved in regulation of important changes occurring during smoltification including regulation of hormone levels, genes important in stress and immune response as well as genes important in ion and water transport across the plasma membrane. MiRNA studies in other vertebrates also supported that some of the orthologs of the Atlantic salmon DE miRNAs have such regulatory roles [38,42,56,57,58,59,60,61,62]. While our results strongly indicate that post-transcriptional modification of gene expression by miRNAs is an important part of the parr-smolt transformation and the following sea water adaption, further functional validation studies are required to confirm, and fully understand, the suggested miRNA-target interactions revealed in this study.

## 5. Conclusions

The results from analysis of miRNA and mRNA expression dynamics in head kidney of Atlantic salmon strongly indicate that post-transcriptional regulation of gene expression by miRNAs is important in smoltification and adaption to sea water. The predicted miRNA target genes, enriched in gene ontology groups hormone biosynthesis, stress management, immune response and ion transport, may be further studied and confirmed by functional validation studies.

## Figures and Tables

**Table 1:** Sampling time points during the experimental period.

Description of Sampling Points		Light ^1^	Temp ^2^	Av. Weight ^3^	Water Type	Sampling ^4^
T1	One day prior to onset of light treatment	24	8	29.4 ± 5.6	Fresh water	Day 0
T2	Halfway through light treatment	12	12	52.6 ± 5.9	Fresh water	Day 47
T3	Three quarters into the light treatment period	24	8	63.9 ± 10.1	Fresh water	Day 67
T4	One day prior to SWT	24	8	72.4 ± 8.7	Fresh water	Day 81
T5	One week after SWT	24	8	63.2 ± 8.5	seawater	Day 88
T6	One month after SWT	24	8	98.4 ± 14.9	seawater	Day 111

^1^ Hours with day light. ^2^ Water temperature in °C. ^3^ Average weight (g) with standard deviation. ^4^ Sampling day during the experiment.

**Table 2:** Relative expression of major expressed family members in Cluster I and II.

No. ^1^	Mature miRNAs	T2 ^2^	T3 ^3^	T4 ^4^	T5 ^5^	T6 ^6^
1	ssa-let-7b-3p	−1.02 *	−0.39	−0.52	−1.29 *	−0.80
2	ssa-miR-20b-5p	−0.61	−0.82	−0.92	−0.98	−1.23 *
3	ssa-miR-30a-3-4-3p	−0.37	−0.54	−0.93	−0.66	−1.05 *
4	ssa-miR-125b-1-3p	−0.74	−1.18 *	−1.51 *	−1.38 *	−1.62 *
5	ssa-miR-130d-2-5p	−0.63	−1.30	−1.30	−1.38 *	−1.26 *
6	ssa-miR-135a-3p	−0.70	−0.46	−0.75	−0.77	−1.63 *
7	ssa-miR-192a-5p	−0.87	−1.67	−4.44 *	−3.16 *	−5.24 *
8	ssa-miR-194a-5p	−2.06	−3.01 *	−4.02 *	−3.22 *	−5.17 *
9	ssa-miR-196a-5p	−0.36	−0.13	−0.89	−0.49	−1.00 *
10	ssa-miR-200ae-3p	−0.69	−3.12 *	−4.40 *	−2.90 *	−5.60 *
11	ssa-miR-200b-3p	−1.54	−2.85 *	−3.91 *	−3.89 *	−5.07 *
12	ssa-miR-203a-3p	0.96	-0.85	−1.25 *	−0.39	−1.16 *
13	ssa-miR-205b-5p	1.29	−0.99	−1.80 *	−1.46 *	−3.05 *
14	ssa-miR-222b-5p	−0.90	−0.63	−0.75	−1.24 *	−1.00
15	ssa-miR-301a-5p	−1.07	−1.59	−2.00 *	−2.37 *	−1.69 *
16	ssa-miR-375-3p	−0.58	−0.40	−1.02 *	−0.07	−0.90
17	ssa-miR-429ab-3p	−0.60	−1.63	−4.08 *	−2.48 *	−5.23 *
18	ssa-miR-430b-5p	−0.84	−0.61	−1.51 *	−1.88 *	−1.71 *
19	ssa-miR-449ab-5p	−0.82	−1.44	−2.57 *	−1.98	−4.44 *
20	ssa-miR-722-3p	−0.26	−0.79	−1.61 *	−0.65	−1.36 *
21	ssa-miR-8162-5p	−0.79	−0.97	−1.20 *	−0.99	−0.79
22	ssa-miR-novel-2-5p	−1.11	−1.48 *	−2.35 *	−1.51 *	−2.42 *
23	ssa-miR-novel-15-3p	−0.78	−1.32 *	−1.55 *	−0.96	−1.02 *
24	ssa-miR-1-3p	−0.05	−1.24	0.15	−1.31 *	−0.99
25	ssa-miR-15b-3p	−0.48	−0.22	−0.61	−1.30 *	−0.22
26	ssa-miR-30a-2-3p	−1.11 *	−0.73	−0.94	−0.97	−0.85
27	ssa-miR-92a-1-2-5p	−1.01 *	−0.21	−0.58	−0.42	−0.18
28	ssa-miR-106b-3p	−0.86	−0.46	−0.97	−1.56 *	−0.45
29	ssa-miR-125a-1-3p	−1.61 *	−0.37	−0.02	−0.26	−0.41
30	ssa-miR-133-4-5p	−0.95	−1.26	−0.72	−1.76	−1.19 *
31	ssa-miR-144-3p	−0.40	−0.10	0.03	−1.53 *	0.69
32	ssa-miR-144-5p	−0.80	−0.38	−0.33	−2.35 *	0.42
33	ssa-miR-301b-5p	−1.17 *	−0.65	−0.26	−1.02 *	−0.38
34	ssa-miR-451-3p	−0.96	−1.01 *	−0.86	−1.68 *	0.27
35	ssa-miR-730a-5p	−0.37	−0.11	0.10	−2.89 *	0.50
36	ssa-miR-2188-3p	−0.30	0.37	0.68	−1.33 *	0.99
37	ssa-miR-novel-12-5p	−0.77	−0.38	−0.69	−1.05 *	−0.29

^1^ Number of DE miRNAs. The table shows only the major expressed mature miRNAs derived from a given precursor in Cluster I (1–23) and Cluster II (24–37) except miRNA-144 where both 5p and 3p were equally expressed. All values are log2 fold changes relative to T1. ^2^ Halfway through light treatment. ^3^ Three quarters into the light treatment period. ^4^ One day prior to seawater transfer (SWT). ^5^ One-week post SWT. ^6^ One-month post SWT. Asterisk (*) indicate expression changes that are significant.

**Table 3:** Relative expression of major expressed family members in Cluster III.

No. ^1^	Mature miRNAs	T2 ^2^	T3 ^3^	T4 ^4^	T5 ^5^	T6 ^6^
1	ssa-miR-17-1-3p	0.36	0.69	0.92	0.78	1.06 *
2	ssa-miR-29b-1-5p	0.84	1.21 *	1.59 *	1.44 *	0.76
3	ssa-miR-100a-2-3p	0.22	0.77	0.98	1.11 *	1.10 *
4	ssa-miR-132-1-2-5p	0.50	0.39	0.57	1.21 *	0.77
5	ssa-miR-137a-3p	0.34	0.23	0.80	1.01 *	0.84
6	ssa-miR-146a-5p	0.58	0.62	0.77	1.28 *	1.00 *
7	ssa-miR-150-3p	0.48	0.56	1.10 *	0.25	0.17
8	ssa-miR-153a-3p	0.51	0.52	0.46	1.08 *	0.95
9	ssa-miR-202b-5p	1.13	0.76	1.18	1.56	2.08 *
10	ssa-miR-204-5p	0.35	0.51	0.72	1.27 *	1.01 *
11	ssa-miR-216a-5p	1.42 *	0.44	0.84	0.13	0.74
12	ssa-miR-217-5p	1.15 *	−0.35	0.44	−0.17	0.40
13	ssa-miR-218a-5p	0.32	0.51	0.79	1.23 *	1.02 *
14	ssa-miR-451-5p	0.44	0.51	0.71	0.27	1.01 *
15	ssa-miR-725-3p	1.46 *	−0.19	−0.39	0.44	0.17
16	ssa-miR-727a-5p	0.70	1.12 *	0.91	0.30	−0.01
17	ssa-miR-8158-3p	0.58	0.52	1.02 *	1.27 *	1.25 *

^1^ Number of DE miRNAs. The table shows only the major expressed mature miRNAs within the same family in Cluster III. All values are log2 fold change relative to T1. ^2^ Halfway through light treatment. ^3^ Three quarters into the light treatment period. ^4^ One day prior to SWT. ^5^ One-week post SWT. ^6^ One-month post SWT. Asterisk (*) indicate expression changes that are significant.

**Table 4:** DE miRNAs in Cluster I and III with their negatively correlated predicted target genes.

No.	Gene Symbols ^1^	DE miRNA ^2^	No.	Gene Symbols ^1^	DE miRNA ^2^
1	*cxa1*	ssa-miR-30e-3-3p **ssa-let-7b-3p** ssa-miR-194a-3p	22	*slc16a7*	**ssa-miR-204-5p** **ssa-miR-217-5p** **ssa-miR-8158-3p**
2	*apoa1bp*	**ssa-miR-novel-15-3p**	23	*acy3*	**ssa-miR-204-5p**
3	*star, paralog 1*	ssa-miR-30e-3-3p	24	*rmdn2*	**ssa-miR-216a-5p**
4	*star, paralog 2*	**ssa-miR-205b-5p**	25	*cdo1*	**ssa-miR-216a-5p**
5	*fdx1l*	**ssa-miR-722-3p**	26	*slc15a2*	**ssa-miR-217-5p**
6	*spink1*	**ssa-miR-196a-5p** ssa-miR-196b-5p	27	*loc106561979*	**ssa-miR-217-5p** **ssa-miR-727a-5p**
7	*ptdss1*	**ssa-miR-17-1-3p**	28	*aqp8*	**ssa-miR-217-5p**
8	*agtrap*	**ssa-miR-17-1-3p**	29	*igfbp5*	**ssa-miR-217-5p**
9	*gulp1*	**ssa-miR-17-1-3p**	30	*gucy2f*	**ssa-miR-217-5p** **ssa-miR-725-3p**
10	*spint2*	**ssa-miR-17-1-3p** **ssa-miR-217-5p**	31	*gat2*	**ssa-miR-218a-5p**
11	*grb14*	**ssa-miR-17-1-3p** **ssa-miR-204-5p** **ssa-miR-218a-5p**	32	*slco1a2*	**ssa-miR-218a-5p** ssa-miR-146a-3-3p
12	*trhde.2*	**ssa-miR-137a-3p** ssa-miR-29c-5p	33	*gfra1*	**ssa-miR-218a-5p** **ssa-miR-727a-5p**
13	*lpl*	**ssa-miR-137a-3p**	34	*loc106610933*	**ssa-miR-150-3p**
14	*epn*	**ssa-miR-137a-3p** ssa-miR-29b-3p ssa-miR-29a-3p	35	*npt2a*	ssa-miR-29c-5p
15	*pnmt*	**ssa-miR-146a-5p** ssa-miR-146b-5p	36	*prss23*	ssa-miR-29b-3p ssa-miR-29a-3p
16	*nrip2*	**ssa-miR-146a-5p** **ssa-miR-217-5p** **ssa-miR-725-3p** ssa-miR-202a-5p **ssa-miR-204-5p** ssa-miR-146b-5p	37	*cld3*	ssa-miR-29b-3p ssa-miR-29a-3p
17	*nid1*	ssa-miR-146b-5p	38	*pfkfb1*	**ssa-miR-725-3p**
18	*s47a1*	ssa-miR-146b-5p	39	*sell*	**ssa-miR-725-3p**
19	*acy2*	**ssa-miR-204-5p**	40	*cisd1*	**ssa-miR-725-3p**
20	*sepp1*	**ssa-miR-204-5p**	41	*wt1*	**ssa-miR-727a-5p** ssa-miR-29c-5p
21	*itga6*	**ssa-miR-204-5p**	42	*capsl*	**ssa-miR-132-1-2-5p**

^1^ The gene symbols of predicted targets. No. 1–6 and No. 7–24 were predicted targets of miRNAs in Cluster I and III, respectively. ^2^ The mature DE miRNAs that were predicted to target the genes, the major expressed mature DE miRNAs are shown in bold font.

**Table 5:** Regulation of hormone levels and steroid biosynthetic process.

Gene ^2^	DE-miRNA	miRNA Cluster
*fdx1l*	**ssa-miR-722-3p**	I, decrease
*star,* paralog1 ^1^	ssa-miR-30e-3-3p	I, decrease
*star,* paralog 2 ^1^	**ssa-miR-205b-5p**	I, decrease
*cxa1^1^*	**ssa-let-7b-3p**, ssa-miR-30e-3-3p, ssa-miR-194a-3p	I, decrease

^1^ These genes are also annotated in the stress enrichment group. ^2^ Gene symbol of predicted target genes. The major expressed mature DE miRNAs are bolded.

**Table 6:** Response to stress, inflammation, hypoxia, and Myeloid cell activation in immune response.

Gene ^2^	DE-miRNA	miRNA Cluster
*agtrap*	**ssa-miR-17-1-3p**	III, increase
*lpl*	**ssa-miR-137a-3p**	III, increase
*pfkfb1*	**ssa-miR-725-3p**	III, increase
*cdo1*	**ssa-miR-216a-5p**	III, increase
*cld3*	ssa-miR-29a-3p, ssa-miR-29b-3p	III, increase
*sepp1*	**ssa-miR-204-5p**	III, increase
*epn*	**ssa-miR-137a-3p**, ssa-miR-29a-3p, ssa-miR-29b-3p	III, increase
*sell*	**ssa-miR-725-3p**	III, increase
*star,* paralog1 ^1^	ssa-miR-30e-3-3p	I, decrease
*star,* paralog 2 ^1^	**ssa-miR-205b-5p**	I, decrease
*cxa1* ^1^	**ssa-let-7b-3p**, ssa-miR-30e-3-3p, ssa-miR-194a-3p	I, decrease

^1^ These genes are also annotated in the hormone enrichment group. ^2^ gene symbol of predicted target genes. The major expressed mature DE miRNAs are bolded.

**Table 7:** Ion transport and regulation of ion transport.

Gene ^1^	DE-miRNA	miRNA Cluster
*npt2a*	ssa-miR-29c-5p	III, increase
*s47a1*	ssa-miR-146b-5p	III, increase
*slc16a7*	**ssa-miR-204-5p, ssa-miR-217-5p, ssa-miR-8158-3p**	III, increase
*slco1a2*	ssa-miR-146a-3-3p, **ssa-miR-218a-5p**	III, increase
*gat2*	**ssa-miR-218a-5p**	III, increase
*slc15a2*	**ssa-miR-217-5p**	III, increase
*spink1*	**ssa-miR-196a-5p**, ssa-miR-196b-5p	I, decrease

^1^ Gene symbol of predicted target genes. The major expressed mature DE miRNAs are bolded.

## Supplementary Materials

The following are available online at https://www.mdpi.com/2073-4425/11/9/1059/s1. Figure S1: The experimental fish trial and sampling time points; Figure S2: Distribution of sequence length in all samples after size filtering and adapter removal of reads; Table S1: Enriched GO biological process and cellular locations of negatively correlated DE genes; Table S2: Overview of head kidney samples with their reads and accession numbers; Table S3: List of 71 DE miRNAs with log2-foldchange and their mature sequences; Table S4: List of DE genes obtained by microarray analyses; Table S5: List of DE genes negatively correlated to miRNAs in Cluster I and III; Table S6: All target genes of 71 DE miRNAs; Table S7: Negatively correlated target genes of miRNAs in Cluster I and III.
