# Supplementary material for: Characterization of Differentially Expressed miRNAs and Their Predicted Target Transcripts during Smoltification and Adaptation to Seawater in Head Kidney of Atlantic Salmon
Source: Genes (Basel). 2020 Sep 8;11(9):1059. doi: 10.3390/genes11091059 (PMC7565298; doi:10.3390/genes11091059)
Supplement: Supplementary file 1 [file genes-11-01059-s001.zip › Supplemental file S1.docx]

**Supplemental file S1.** The experimental fish trial and sampling time points


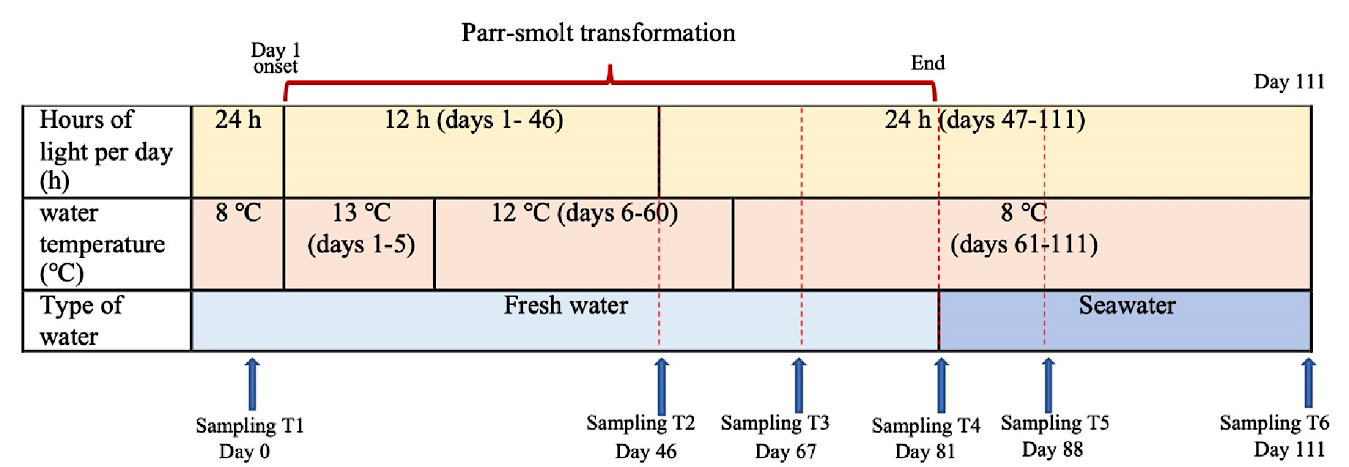


Conditions during light treatment/smoltification and seawater transfer. The blue arrow lines indicate the time of samplings. Sampling T1 was collected one day prior to the initiation of light treatment (day -1). Sampling T2, T3, T4, T5 and T6 were collected on experiment day 46, 67, 81, 88 and 111, respectively.
