# Supplementary material for: Characterization of Differentially Expressed miRNAs and Their Predicted Target Transcripts during Smoltification and Adaptation to Seawater in Head Kidney of Atlantic Salmon
Source: Genes (Basel). 2020 Sep 8;11(9):1059. doi: 10.3390/genes11091059 (PMC7565298; doi:10.3390/genes11091059)

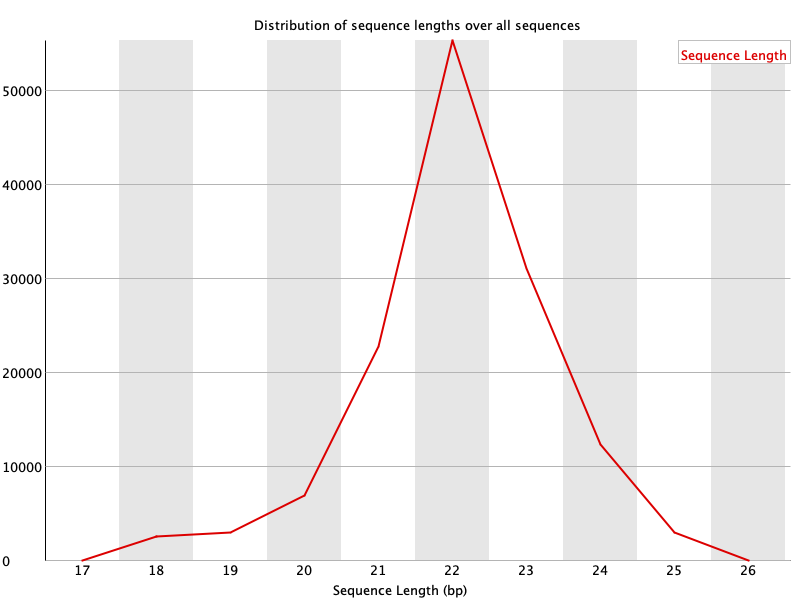

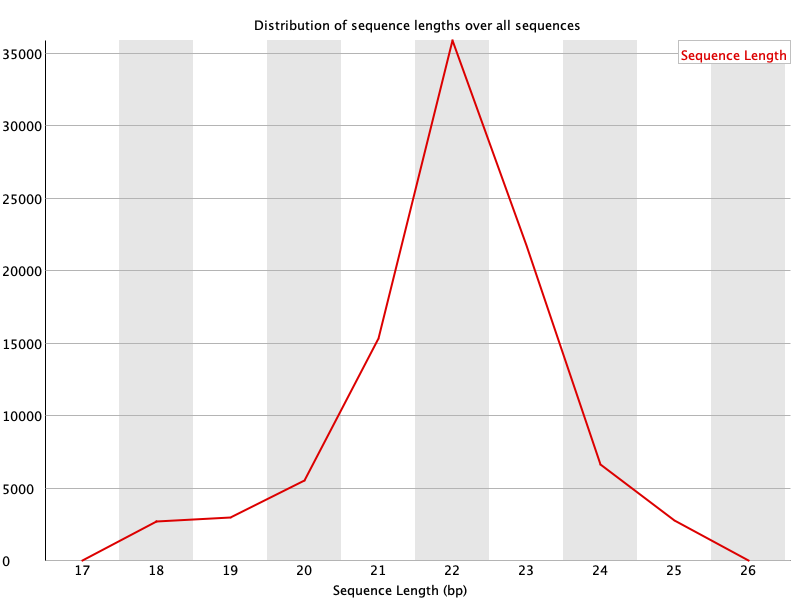

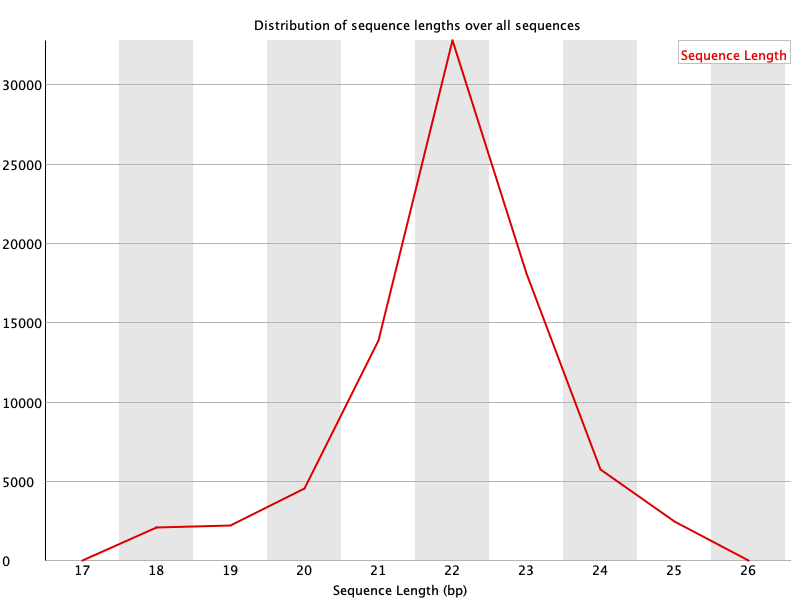

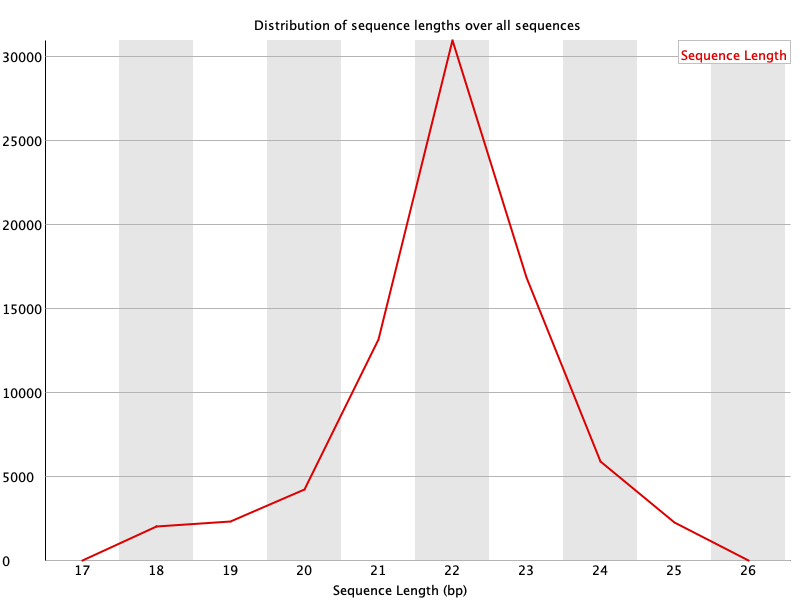

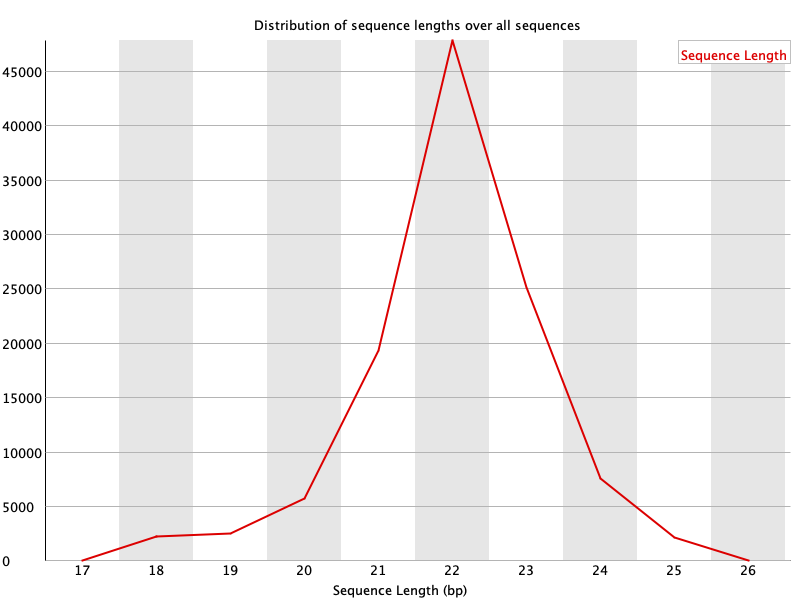

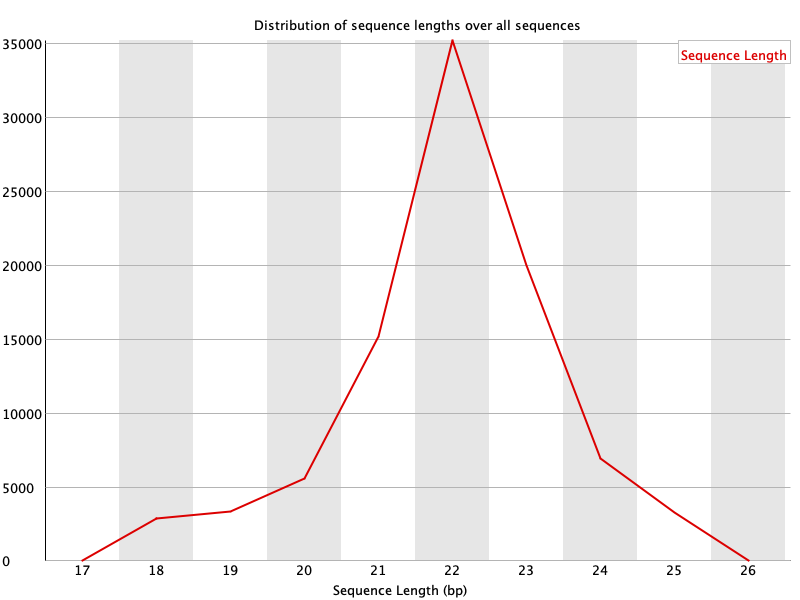

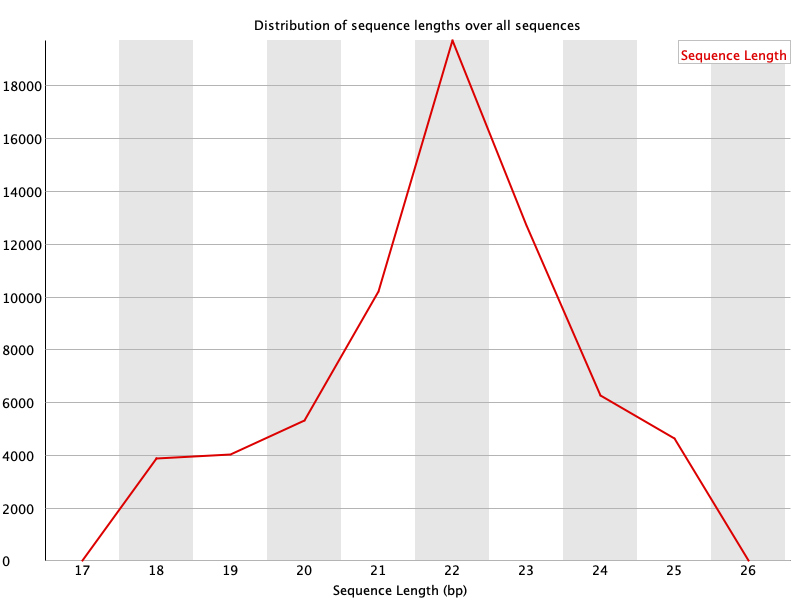

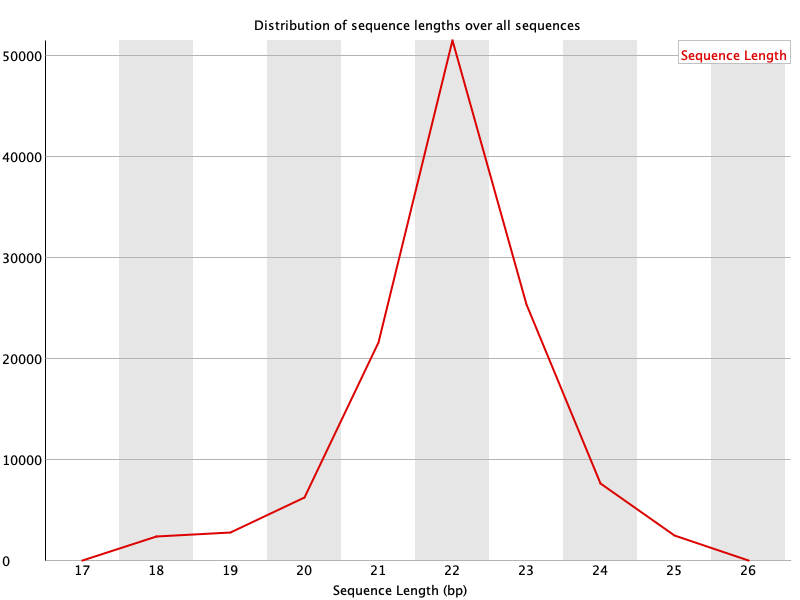


**Supplemental file S3**. Distribution of sequence length in all samples after size filtering and adapter removal of reads. Samples are given from left to right and from top to bottom (sample 1 on top left and sample 48 on last page to bottom right).


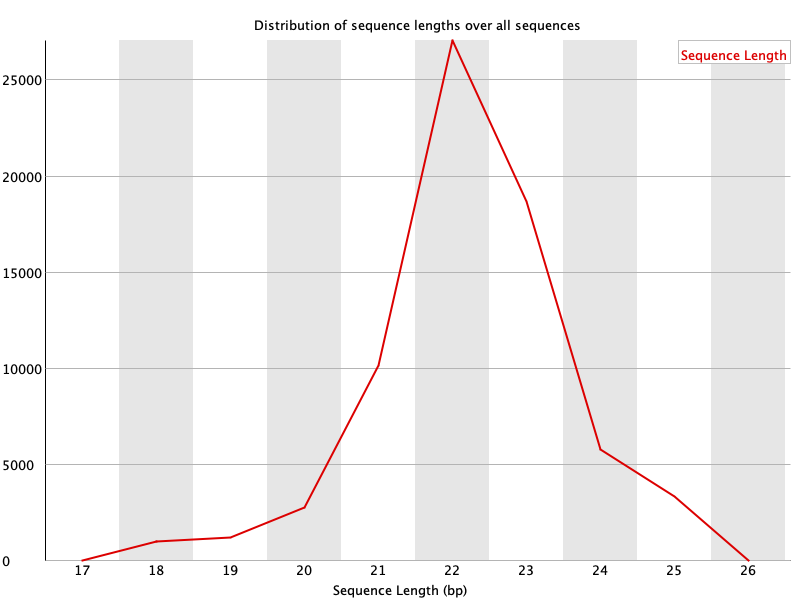

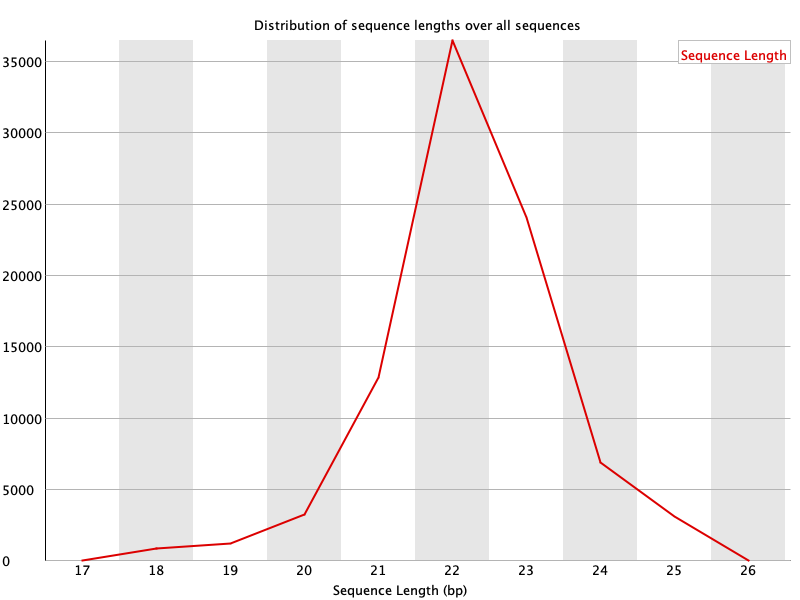

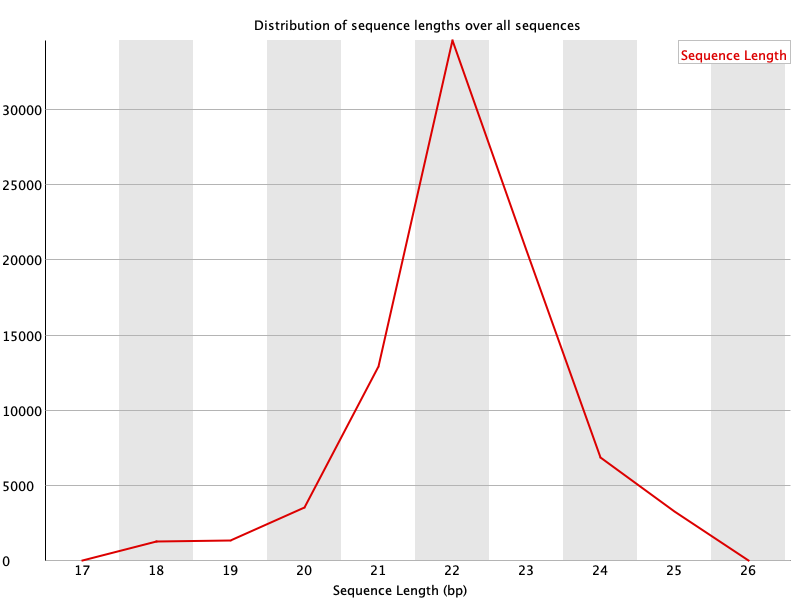

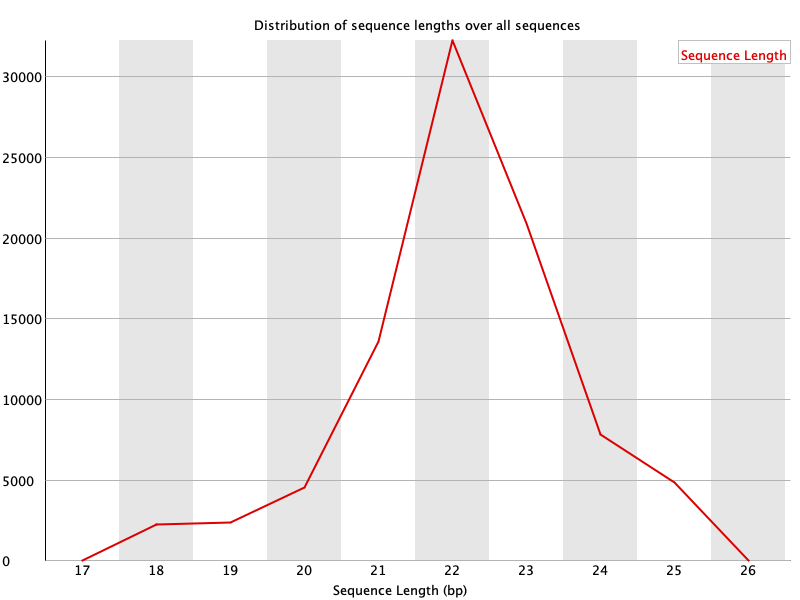

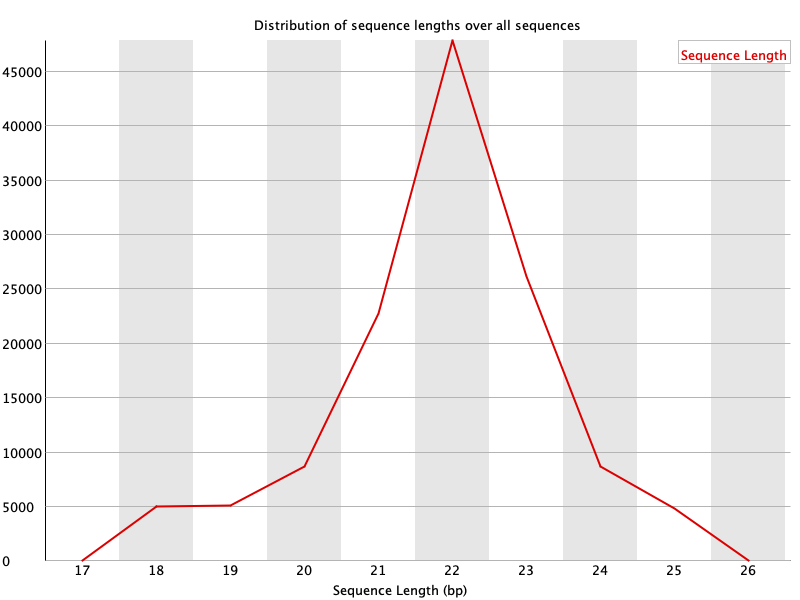

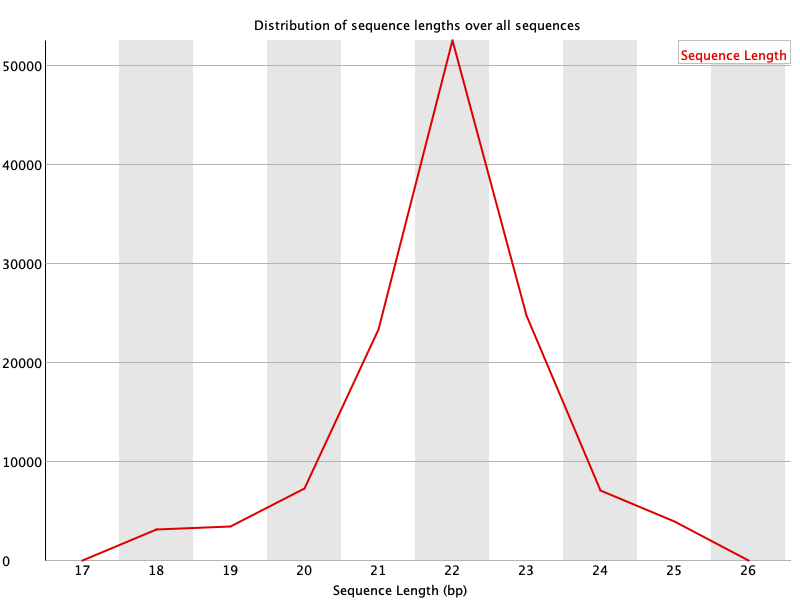

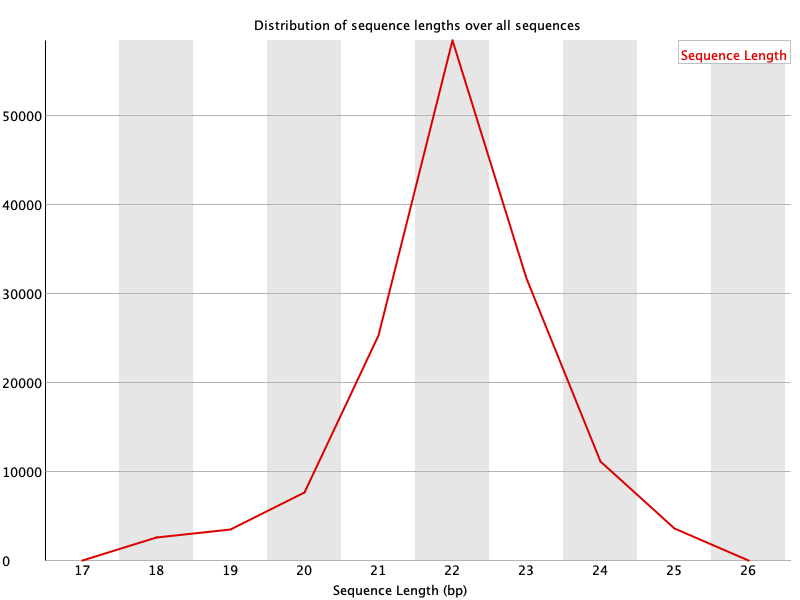

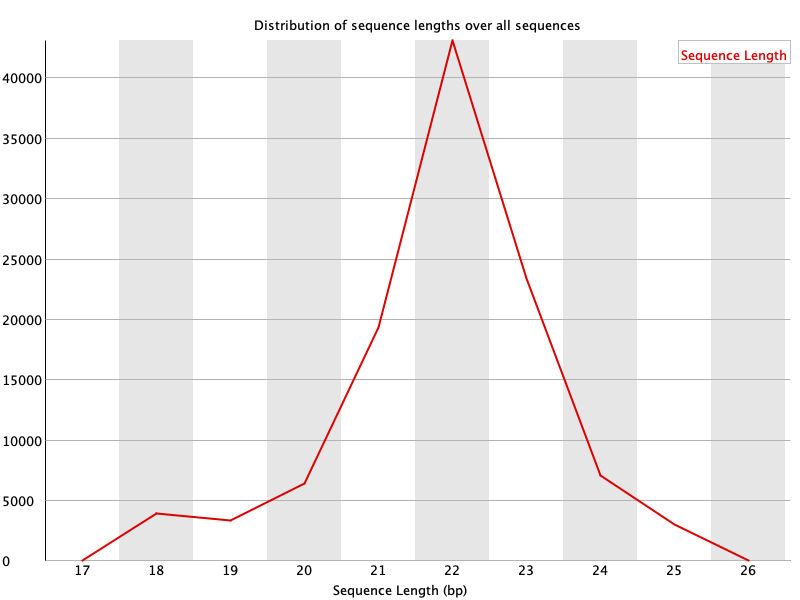


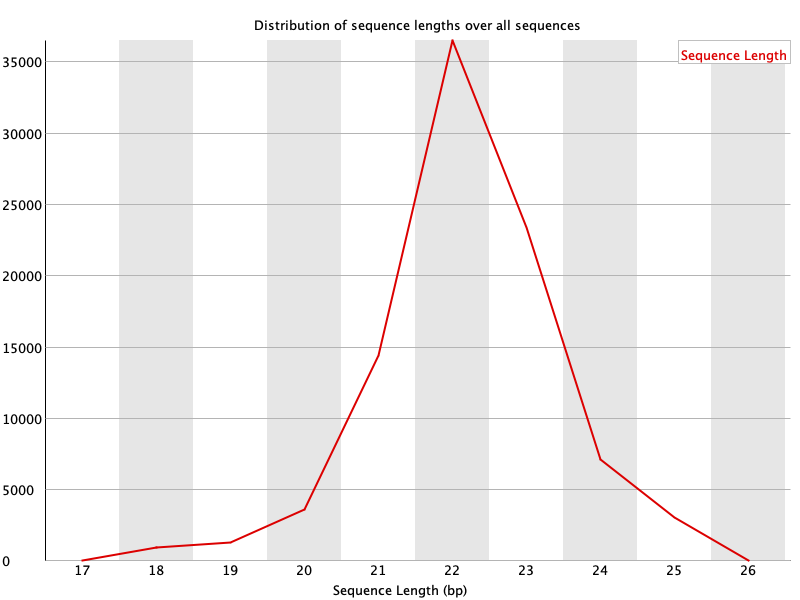

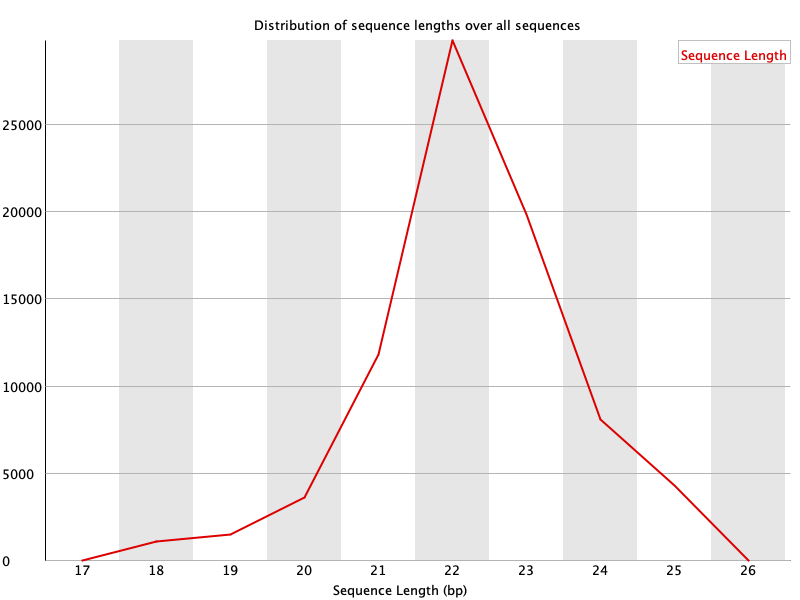

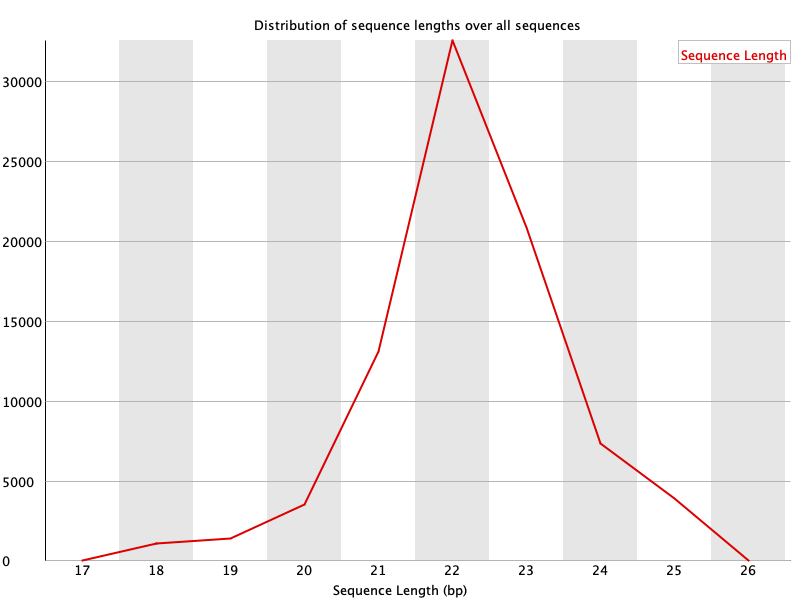

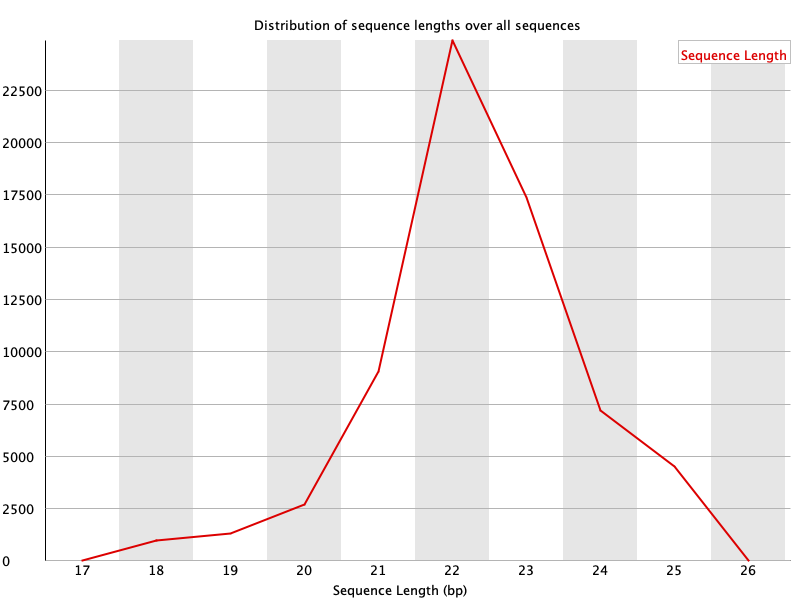

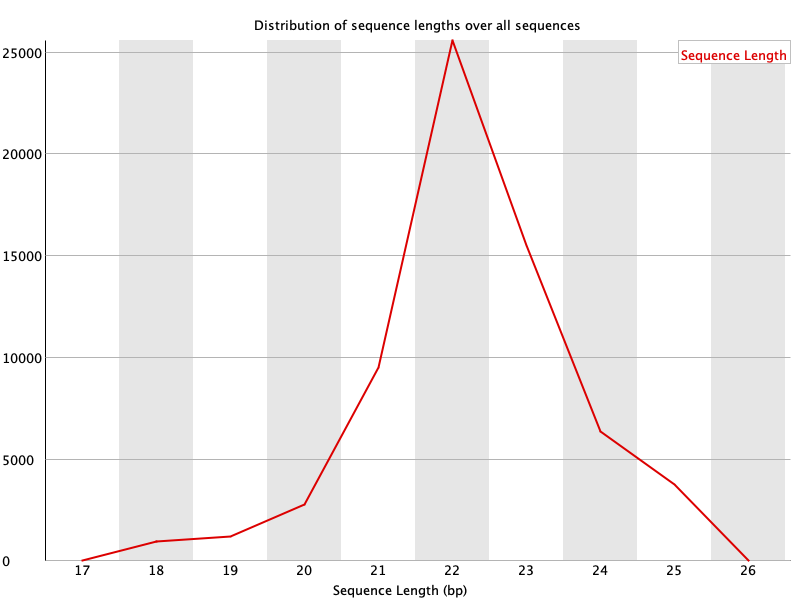

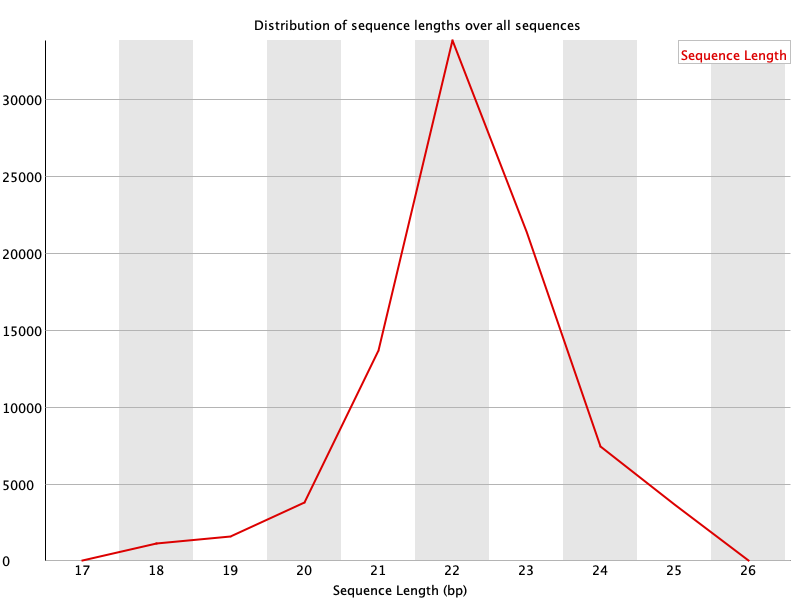

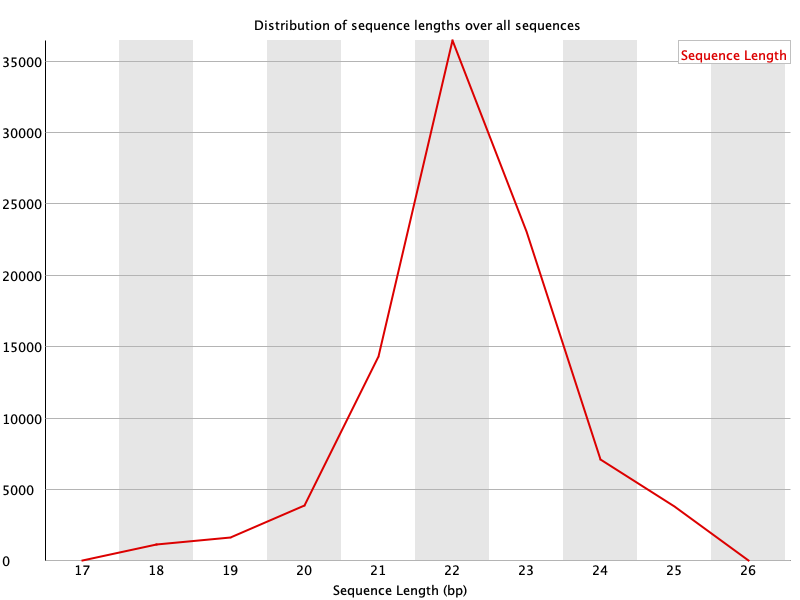

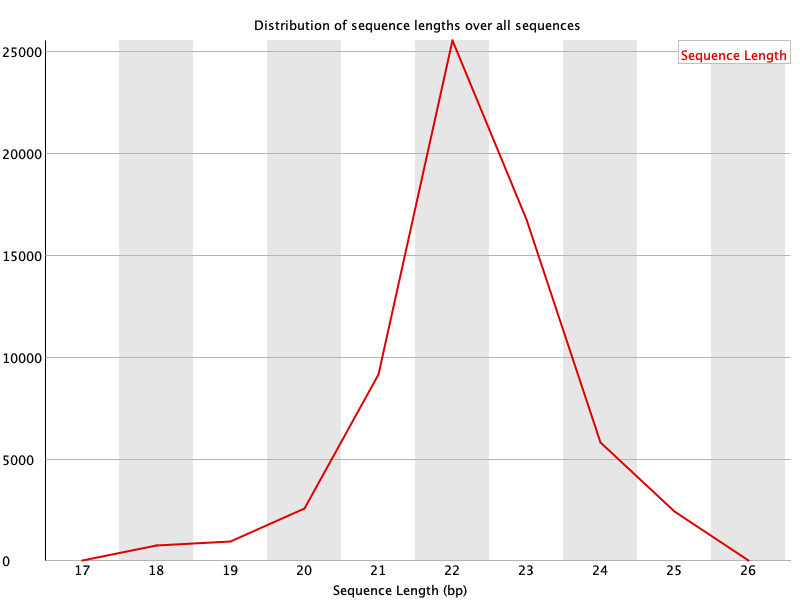


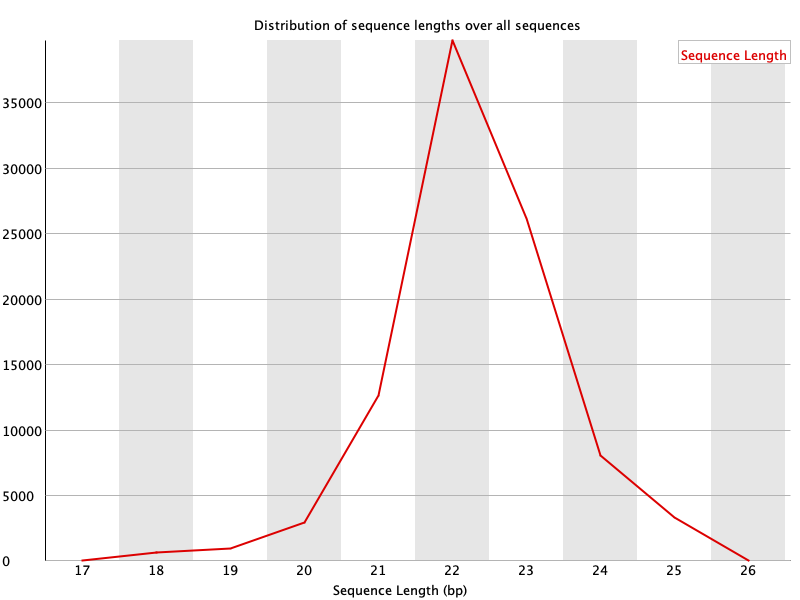

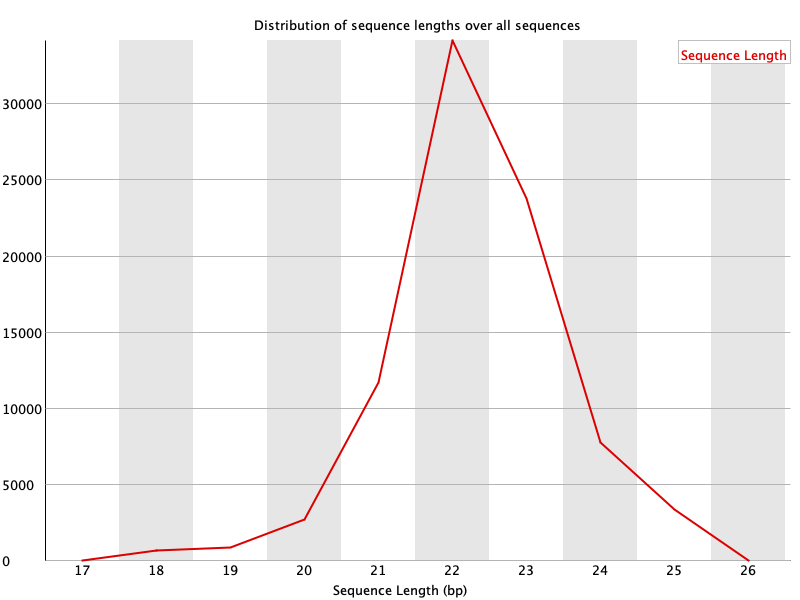

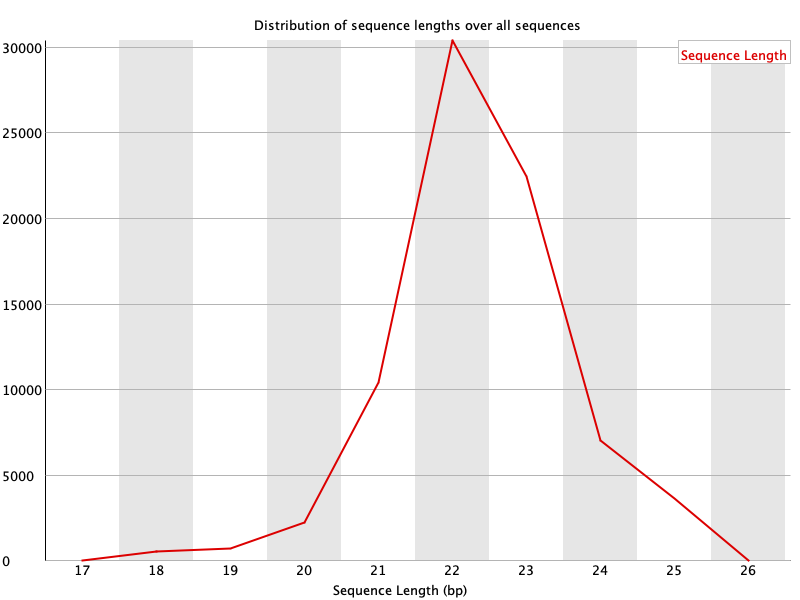

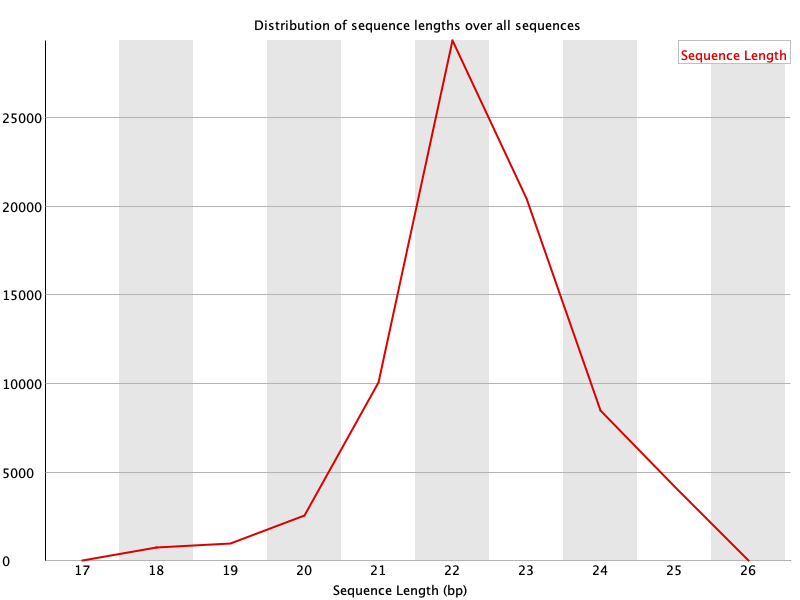

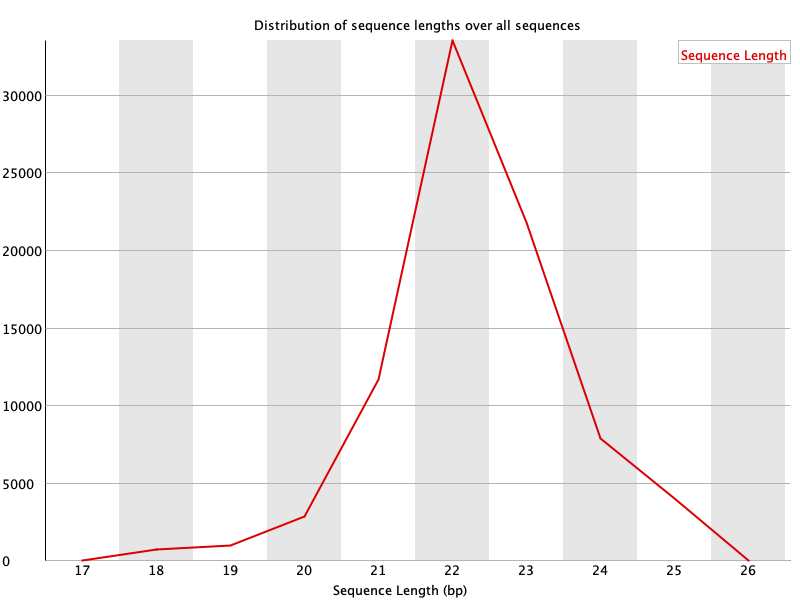

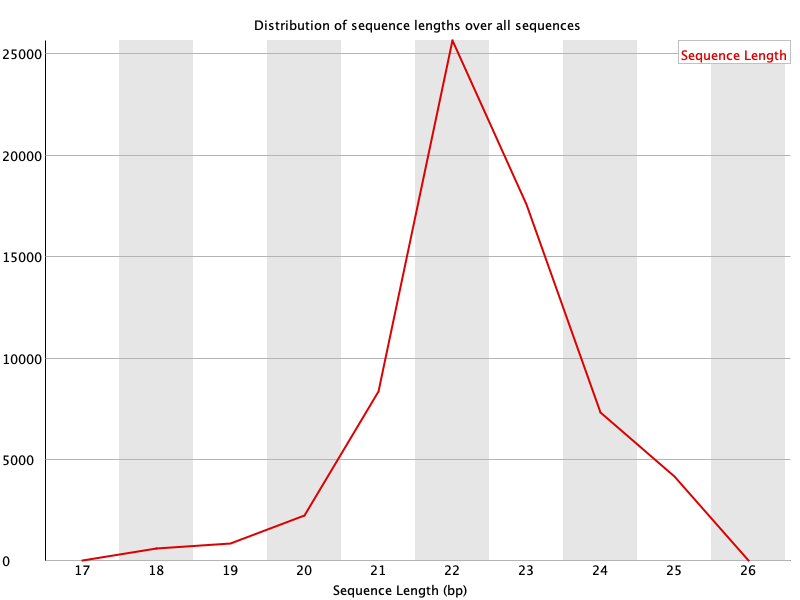

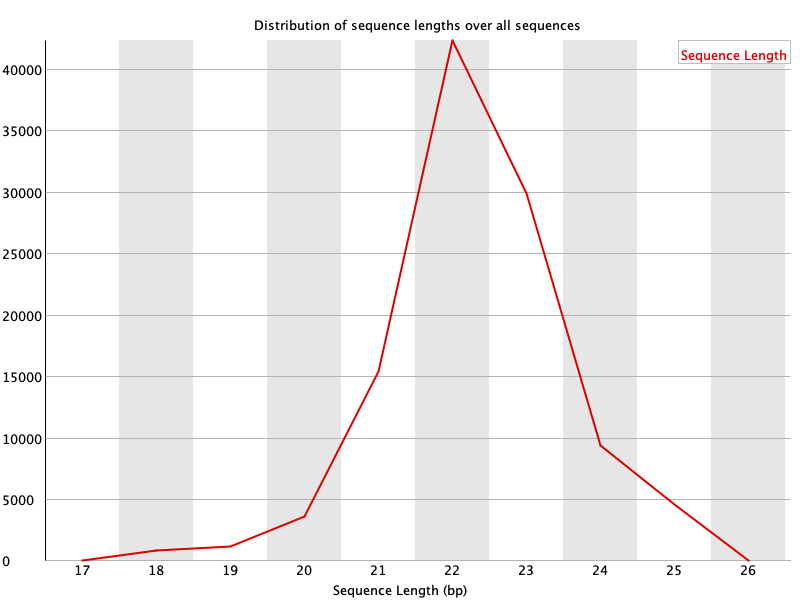

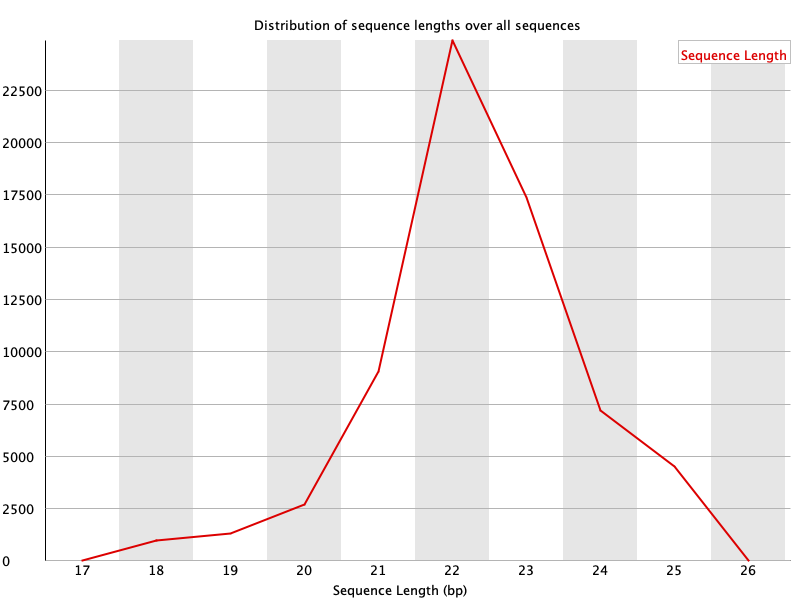


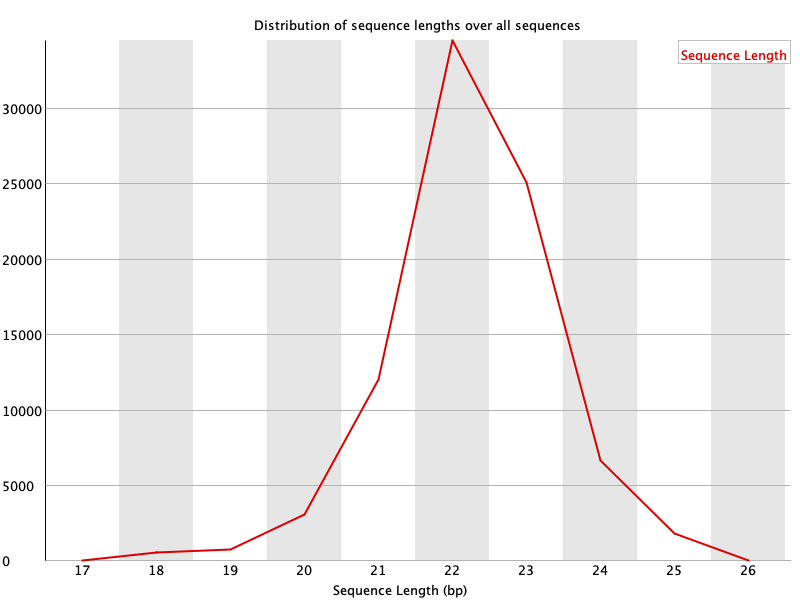

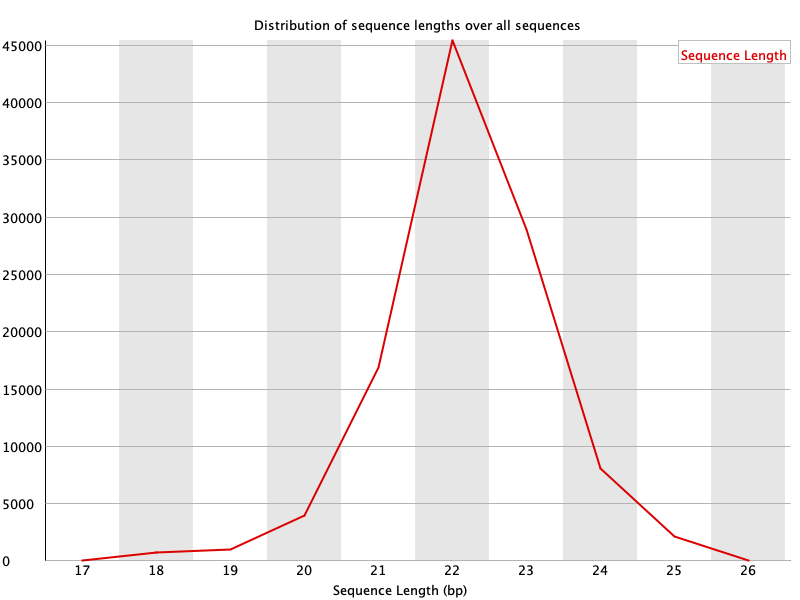

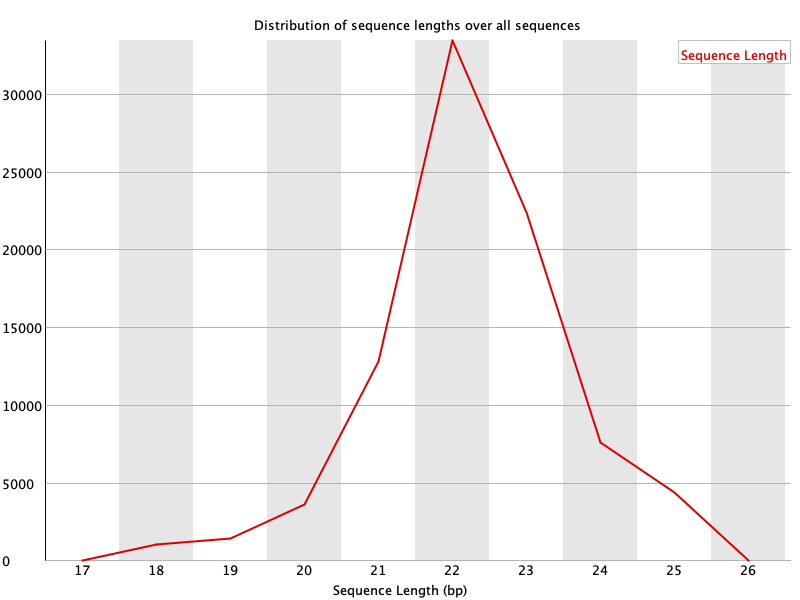

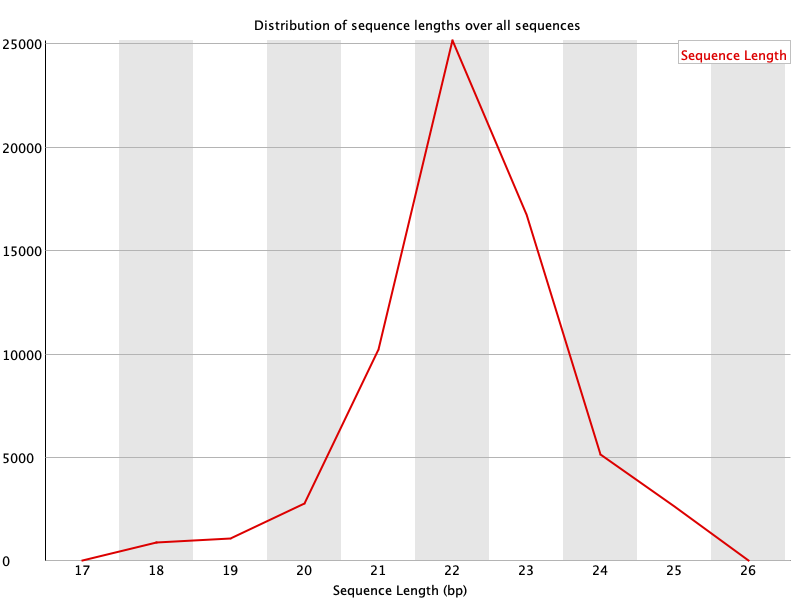

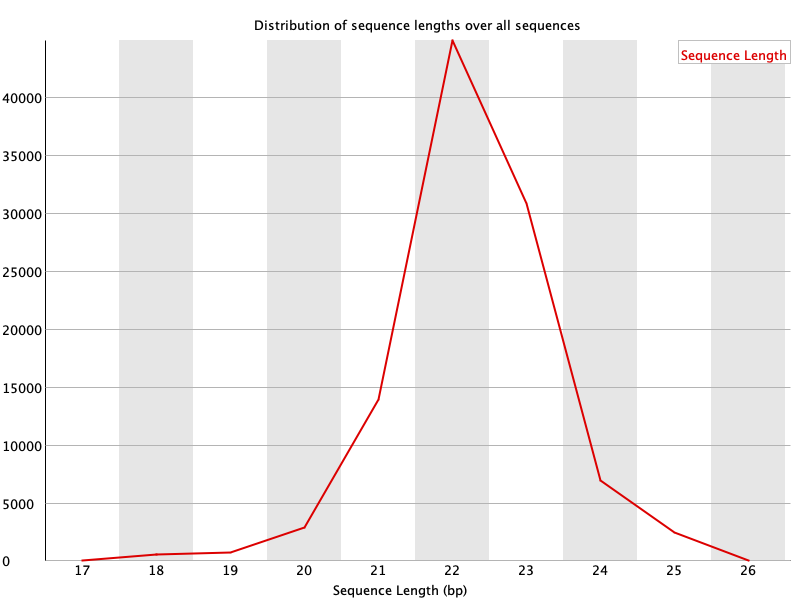

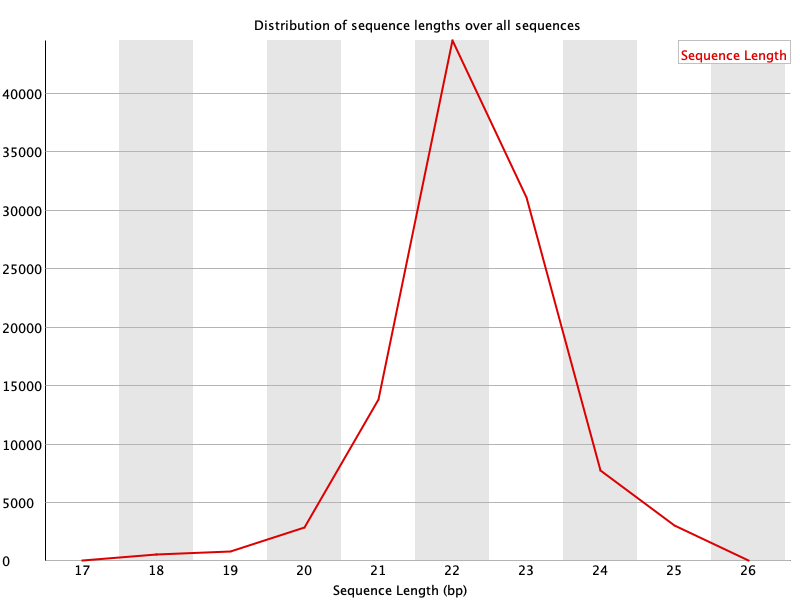

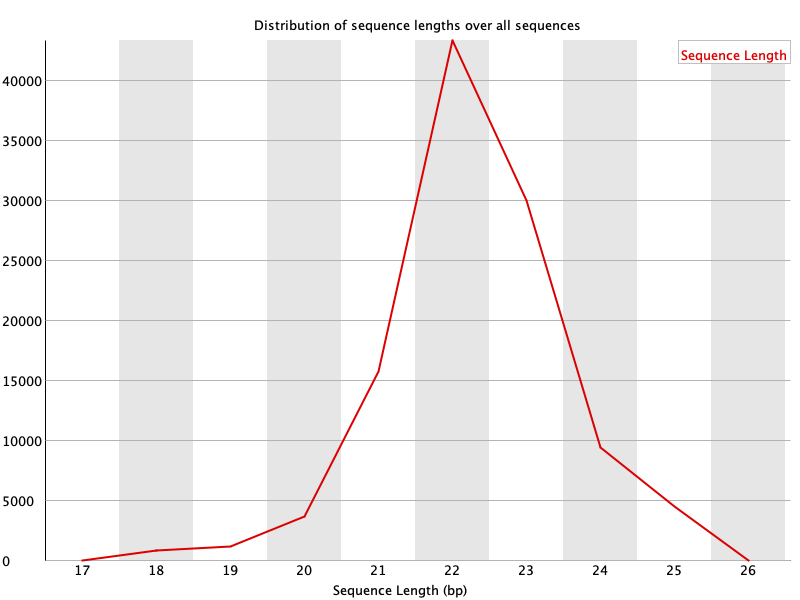

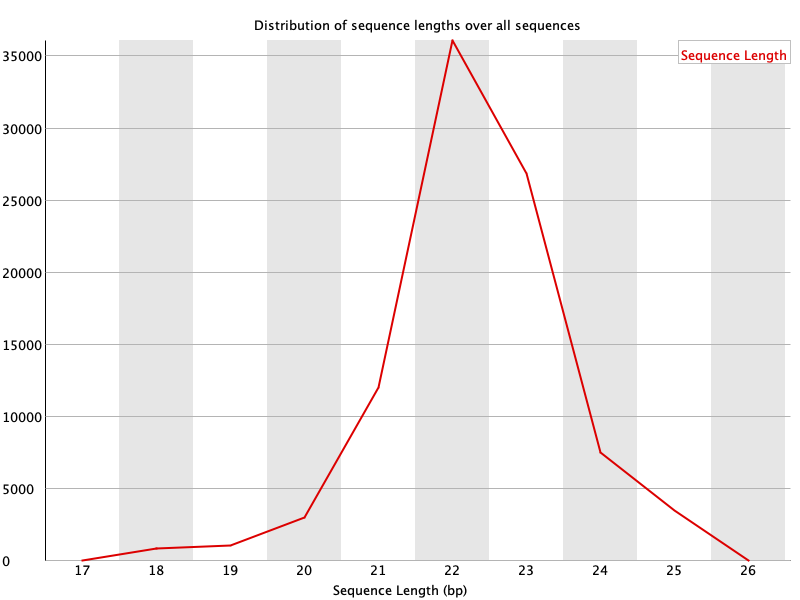


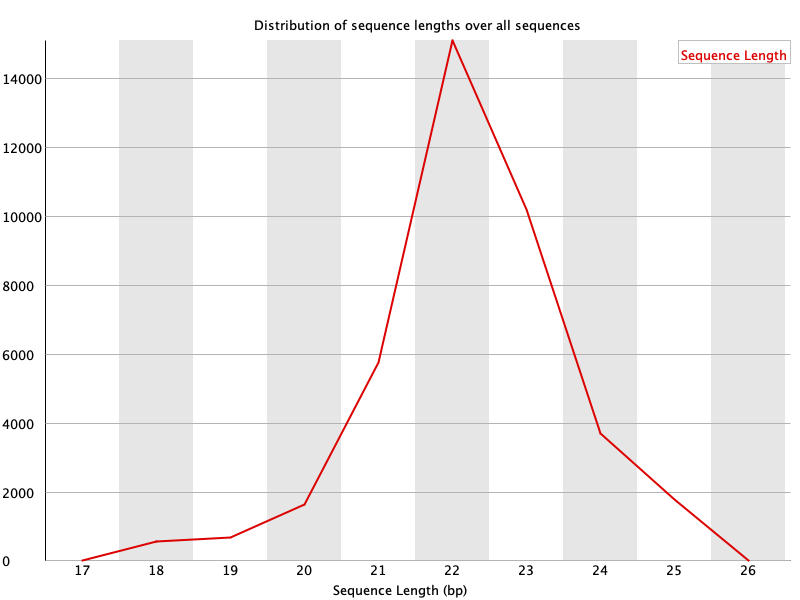

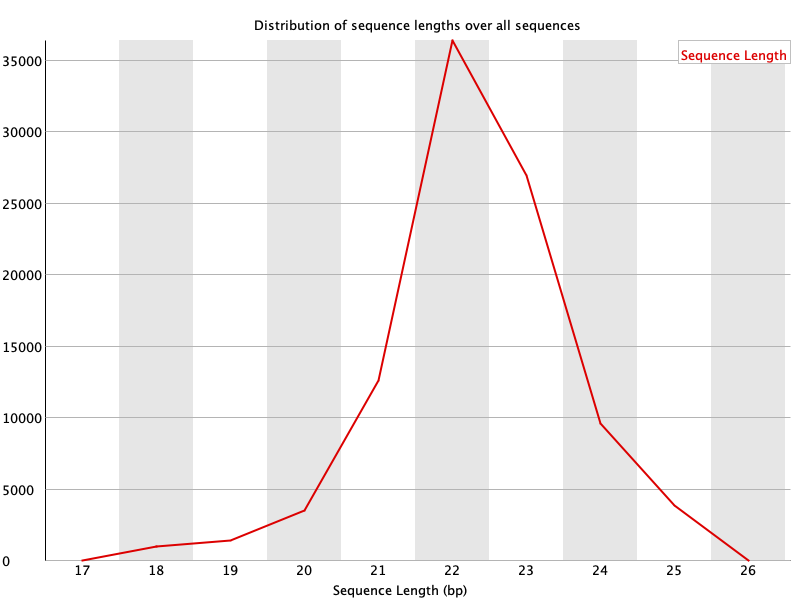

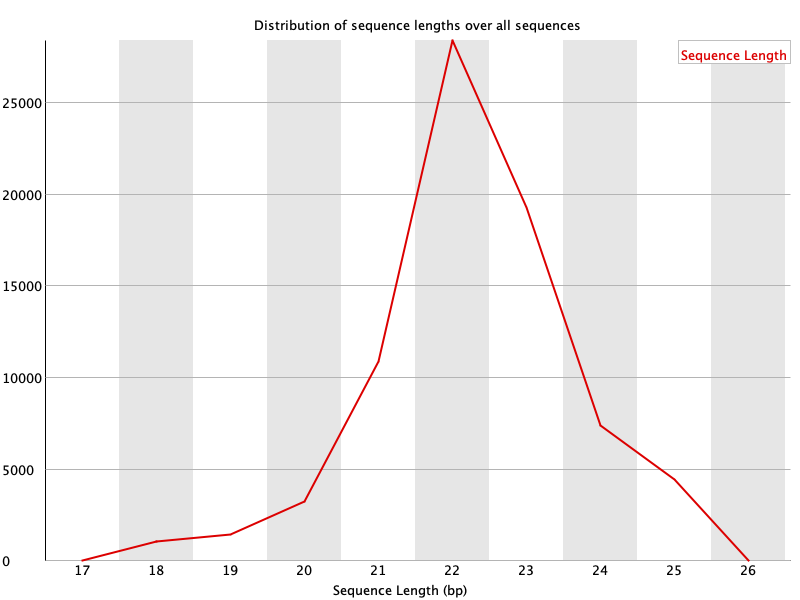

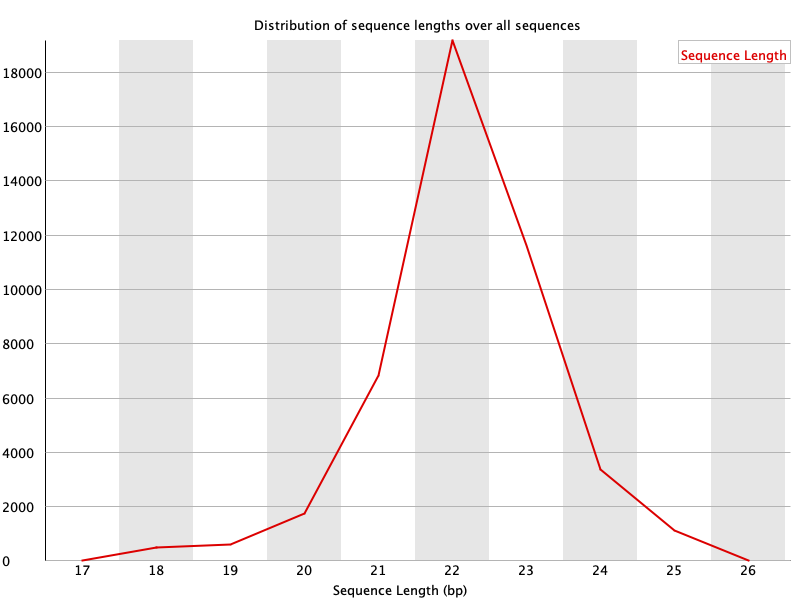

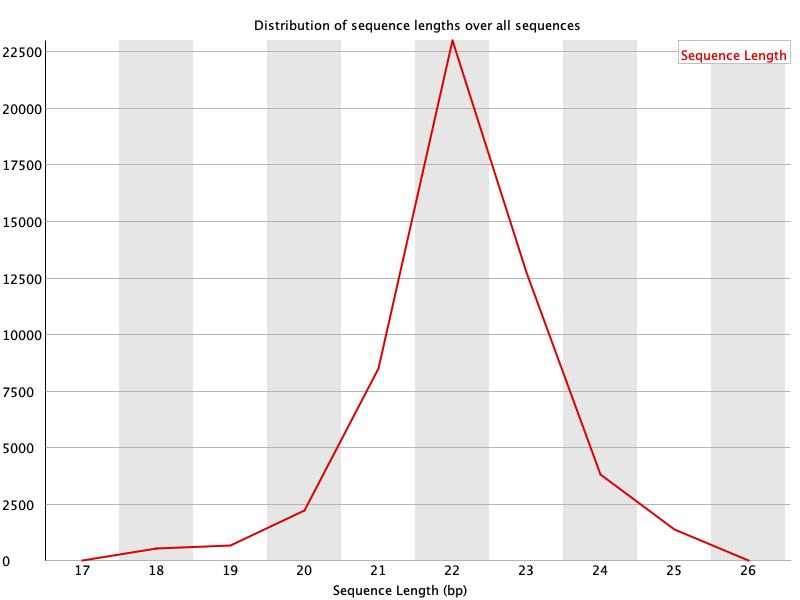

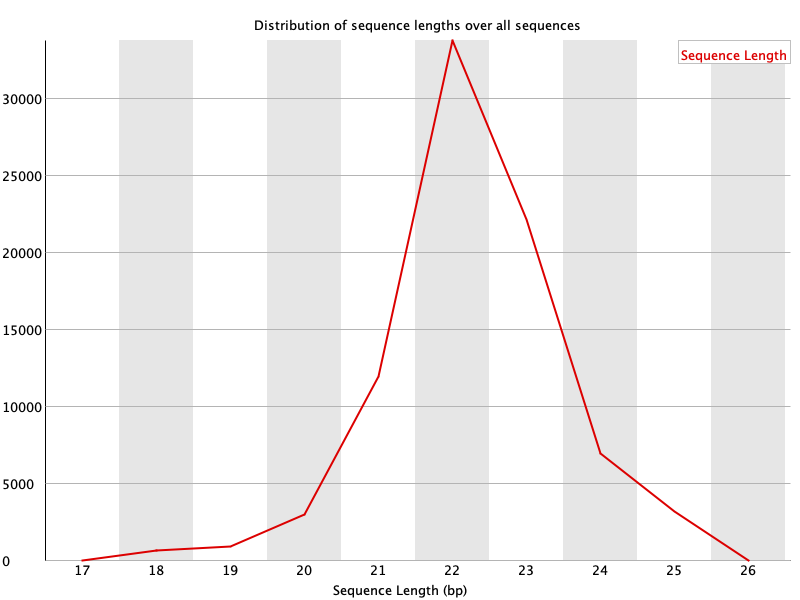

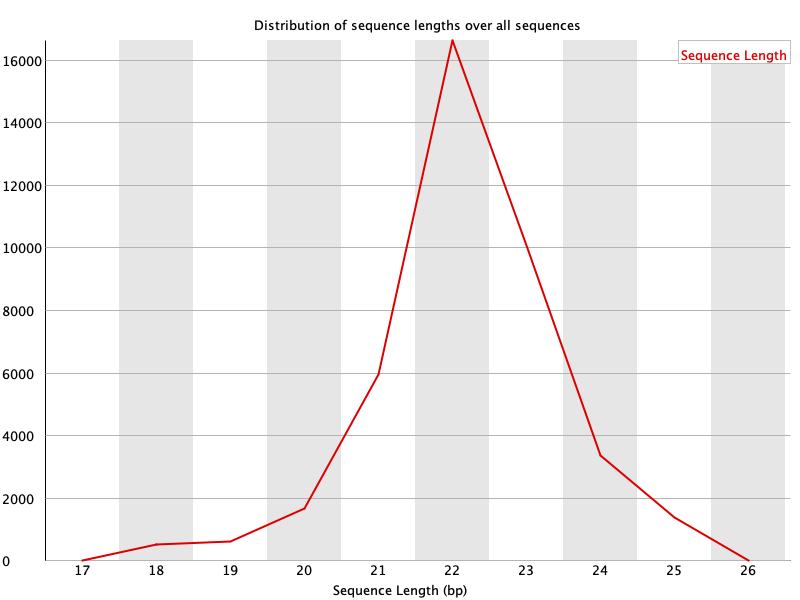

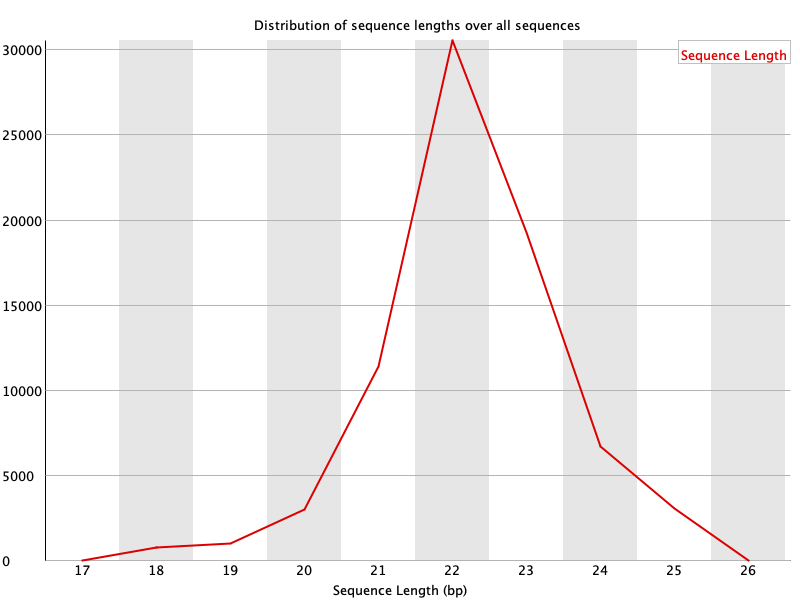

Supplement: Supplementary file 1 [file genes-11-01059-s001.zip › Supplemental file S3.docx]
